# Mesophilic and Thermophilic Conditions Select for Unique but Highly Parallel Microbial Communities to Perform Carboxylate Platform Biomass Conversion

**DOI:** 10.1371/journal.pone.0039689

**Published:** 2012-06-22

**Authors:** Emily B. Hollister, Andrea K. Forrest, Heather H. Wilkinson, Daniel J. Ebbole, Susannah G. Tringe, Stephanie A. Malfatti, Mark T. Holtzapple, Terry J. Gentry

**Affiliations:** 1 Department of Soil and Crop Sciences, Texas A&M University, College Station, Texas, United States of America; 2 Department of Pathology and Immunology, Baylor College of Medicine, Houston, Texas, United States of America; 3 Department of Chemical Engineering, Texas A&M University, College Station, Texas, United States of America; 4 Department of Plant Pathology and Microbiology, Texas A&M University, College Station, Texas, United States of America; 5 Joint Genome Institute, Walnut Creek, California, United States of America; 6 Department of Pathology, Texas Children's Hospital, Houston, Texas, United States of America; Argonne National Laboratory, United States of America

## Abstract

The carboxylate platform is a flexible, cost-effective means of converting lignocellulosic materials into chemicals and liquid fuels. Although the platform's chemistry and engineering are well studied, relatively little is known about the mixed microbial communities underlying its conversion processes. In this study, we examined the metagenomes of two actively fermenting platform communities incubated under contrasting temperature conditions (mesophilic 40°C; thermophilic 55°C), but utilizing the same inoculum and lignocellulosic feedstock. Community composition segregated by temperature. The thermophilic community harbored genes affiliated with Clostridia, Bacilli, and a *Thermoanaerobacterium* sp, whereas the mesophilic community metagenome was composed of genes affiliated with other Clostridia and Bacilli, Bacteriodia, γ-Proteobacteria, and Actinobacteria. Although both communities were able to metabolize cellulosic materials and shared many core functions, significant differences were detected with respect to the abundances of multiple Pfams, COGs, and enzyme families. The mesophilic metagenome was enriched in genes related to the degradation of arabinose and other hemicellulose-derived oligosaccharides, and the production of valerate and caproate. In contrast, the thermophilic community was enriched in genes related to the uptake of cellobiose and the transfer of genetic material. Functions assigned to taxonomic bins indicated that multiple community members at either temperature had the potential to degrade cellulose, cellobiose, or xylose and produce acetate, ethanol, and propionate. The results of this study suggest that both metabolic flexibility and functional redundancy contribute to the platform's ability to process lignocellulosic substrates and are likely to provide a degree of stability to the platform's fermentation processes.

## Introduction

As energy demands place increasing pressure on global fuel reserves, the need to develop stable, renewable alternatives to fossil fuels continues to become more urgent. Biomass-based fuels are expected to help offset these demands and, in some cases, are mandated to do so [1], [2]. For example, the US National Renewable Fuel Standard calls for the volume of renewable fuel blended into US transportation fuels to increase from 9 billion gallons in 2008 to 36 billion gallons by 2022 [3].

**Figure 1:**
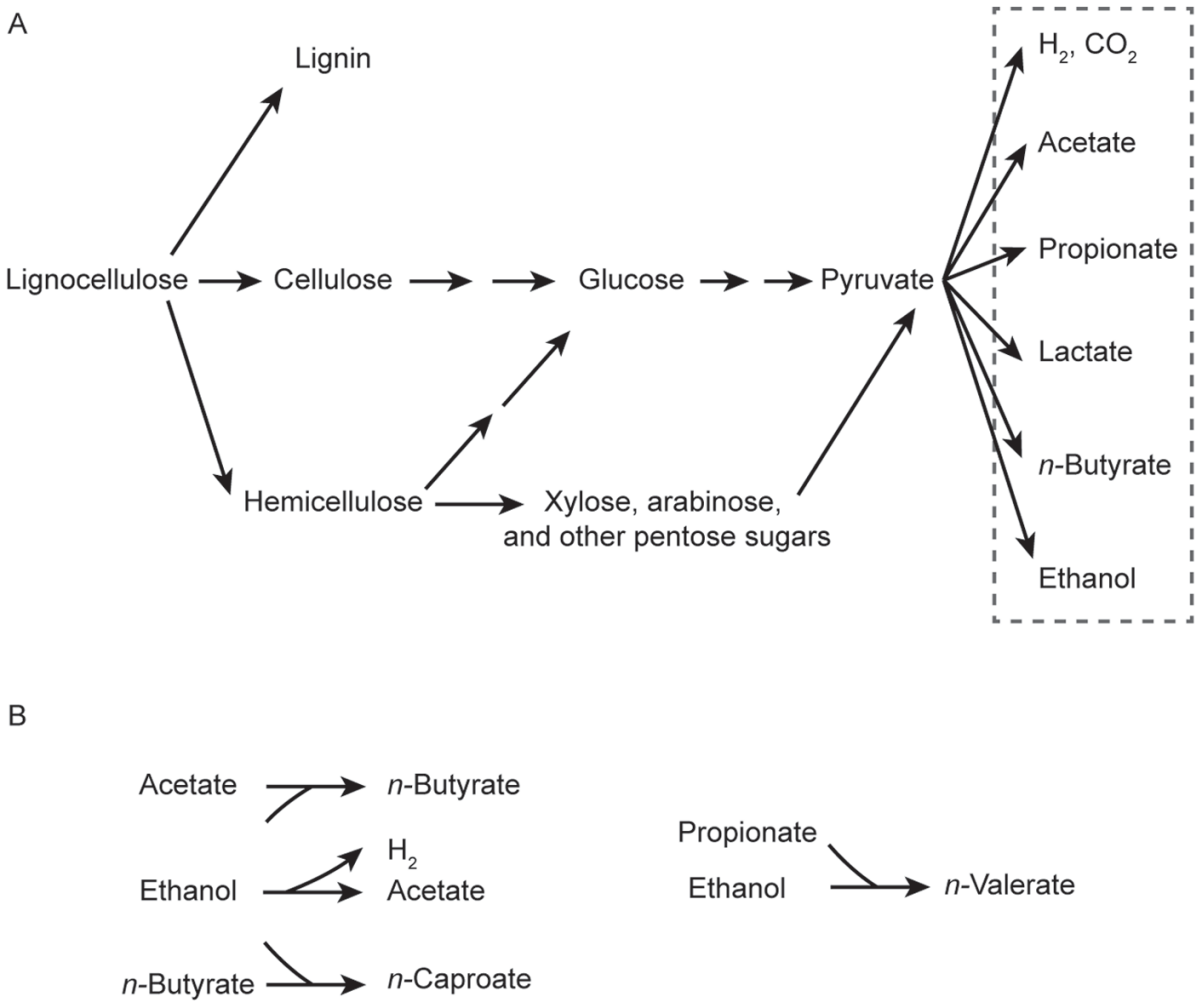
Generalized pathways underlying the conversion of lignocellulose to short chain fatty acids in the carboxylate platform. A) During primary fermentation, pentose and hexose sugars are converted into pyruvate, which may be converted downstream into a variety of primary products (outlined in gray). B) These primary products may undergo secondary fermentation, including chain elongation with ethanol. Multiple arrows indicate that several steps may be involved in the conversion of substrate to product.

Biomass can be converted into liquid fuels using a number of different biorefining approaches, one of which is the carboxylate platform [4], [5]. An alternative to the aseptic fermentation of simple sugars (i.e., ethanol production from sugar or starch) or thermochemical conversion processes, the carboxylate platform operates under non-sterile conditions and uses a mixed community of anaerobic microorganisms to convert lignocellulosic materials into chemicals and liquid fuels [5], [6]. These features allow the platform to be flexible in terms of the variety of feedstocks it can accommodate. Further, it is cost-effective in that it does not require the addition of exogenous enzymes to carry out its conversion and fermentation processes. The platform's primary products are short-chain carboxylates (e.g., acetate, propionate, and *n*-butyrate (Figure 1A)), which can be transformed through downstream chemistry into alcohols, jet fuel, and gasoline. The spectrum of products produced by the platform is temperature dependent [7], [8], [9] and can be varied in response to market demands.

Although it has long been recognized that microbes are integral to the functioning of the carboxylate platform, and a variety of inoculum sources have been evaluated in attempts to improve platform performance [10], the microbial communities that underlie it have long been treated as a black box [4], [11]. Recent work, however, has begun to shed light on them, demonstrating that communities which perform well under the anaerobic, warm, and relatively salty conditions of the carboxylate platform tend to be dominated by bacteria and harbor substantial flexibility with respect to the identities of the taxa involved in the platform's bioconversion processes [12], [13]. Relatively simple consortia dominated by *Clostridium*- and *Bacillus*-like organisms appear to be characteristic of thermophilic fermentations, whereas substantially more diverse consortia enriched in Bacteroidetes, Actinobacteria, and members of the Firmicutes typify mesophilic fermentations [9], [12], [13]. Despite both temperature conditions harboring many *Clostridium*-like organisms, few are shared in common.

The composition of the carboxylate platform communities that have been characterized to date suggests that, like many rumen and gut communities, they operate synergistically, with different portions of each community performing niche metabolic processes that result in the cooperative degradation of materials that would otherwise be difficult for individual species to digest [14], [15], [16]. Although the composition of platform communities provides strong clues regarding the function of their component members, 16S rRNA-based data cannot actually confirm this. It is clear that these communities can convert biomass into carboxylic acids, but the specific means through which they do this (i.e., metabolic pathways), and the degree to which parallel pathways are utilized within and between communities, remain unknown.

Metagenomics, the direct sequencing and analysis of DNA from mixed communities, provides a means through which functional genes may be identified, pathways elucidated, and metabolic strategies compared. Here we present the characterization of two carboxylate platform fermentor metagenomes operating under contrasting temperature conditions, which are known to harbor distinctly different bacterial consortia produce divergent spectra of mixed acid products [9]. The objectives of this study were to identify the similarities and differences shared between these two metagenomes and compare the fermentor metagenomes to those of other well-established lignocellulose-degrading consortia.

## Results

After 16 days' incubation, the mesophilic and thermophilic fermentations resulted in similar rates of biomass conversion, selectivity, yield, and productivity (Table S1); however, the two temperature conditions differed with respect to the abundances of multiple acids within their product spectra (Table 1). Significant differences were observed with respect to the abundances of propionic (C_3_), valeric (C_5_), and caproic (C_6_) acids, each of which was produced in greater quantities by the mesophilic community.

**Table 1:** Distribution and relative abundance (%) of fermentation products following 16 day's incubation under contrasting fermentation temperatures.

	Relative abundance of acid products (%) 1				
Treatment	Acetic (C_2_)	Propionic (C_3_)	Butyric (C_4_)	Valeric (C_5_)	Caproic (C_6_)
40°C incubation	51.63±0.84 ^a^	12.81±2.06 ^a^	28.39±1.64 ^a^	2.60±0.37 ^a^	4.56±0.72 ^a^
55°C incubation	58.69±2.84 ^a^	1.38±0.69 ^b^	39.52±2.54 ^a^	0.42±0.42 ^b^	ND ^b^

1 Values represent the mean of three replicates ± SE, and all isomers of a given volatile fatty acid are summed together.

a, b Within a column, the use of different letters as superscripts indicates a statistically significant difference between fermentation temperatures (*p*<0.05), as determined by Student's t-test.

ND refers to acid products that were not detected.

Shotgun sequencing efforts resulted in the production of more than 2.5 million sequence reads per fermentor library, representing 900 and 588 Mbp of sequence data for the thermophilic and mesophilic metagenomes, respectively (Table 2). A large proportion of these reads assembled successfully into “large” contigs (i.e., ≥1 kb), with one of the largest contigs exceeding 300 kb in length. The degree to which the protein-coding genes contained within each library could be associated with a predicted function, KEGG orthology, or COG category ranged from 45 to 60%, depending on the metric used but tended to be similar between the two metagenomes (Table S2).

**Table 2:** Metagenome summary statistics.

Metagenome	Total number of reads	Total amount of sequence (Mbp)	Number of contigs	Large contigs (≥1 kb)	Longest contig (bp)
55°C	2,129,475	683	23,406	18,329	233,318
40°C	2,261,434	482	29,995	19,452	312,540

**Figure 2:**
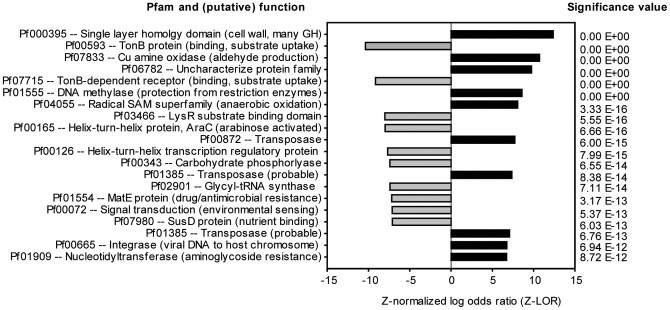
Pfams significantly enriched in the thermophilic (55°C, black) and mesophilic (40°C, gray) metagenomes. Negative Z-normalized log odds ratio values indicate Pfams that were enriched in the mesophilic community, and a complete list of the Pfams that were found to be significantly different between the two communities is provided in Table S3.

Both metagenomes harbored a core set of genes associated with housekeeping, general metabolism, and other functions. Of the approximately 4900 COGs, 5600 EC categories, and 11,900 Pfams evaluated, 11%, 2.5%, 3.3%, respectively, were found to differ significantly between the two fermentor communities. Despite such high levels of similarity, significant differences were detected between the mesophilic and thermophilic metagenomes with respect to the relative abundances of multiple Pfams (Figure 2), COGs, and enzymes. These included the enrichment of genes related to substrate binding, arabinose metabolism, and the degradation of oligosaccharides in the mesophilic metagenome, as well as the enrichment of genes related to the uptake of cellobiose and transfer of genetic material (i.e., transposases, integrases) in the thermophilic metagenome. Complete lists of the functions that were found to differ significantly between the metagenomes are provided in Tables S3, S4, and S5.

Glycosyl hydrolases, families of enzymes key to the degradation of carbohydrate molecules, were well represented in the fermenter metagenomes (Table 3). A total of 1314 GH were identified in the thermophilic metagenome, and 3387 GH were identified in the mesophilic metagenome, representing 0.45 and 0.6% of the protein coding genes identified in each community, respectively. The GH families detected represent known carbohydrate-active enzymes, including cellulases, endohemicellulases, debranching enzymes, and oligosaccharide-degrading enzymes. Each of the GH families detected in the thermophilic fermentor metagenome was also present in the mesophilic community, but significant differences were found with respect to the relative abundances of several. Of particular note were the enrichments of GH48, a family of cellobiohydrolases, in the thermophilic metagenome and GH43, a family of arabinose- and xylose-degrading enzymes, in the mesophilic metagenome. The fermentor metagenomes resembled other well-characterized lignocellulose-degrading metagenomes [14], [15], [16], [17] (Table 3), with the exceptions that the carboxylate platform metagenomes tended to be enriched with respect to GH 48 and depleted with respect to the α-L-rhamnosidase associated with GH 78.

**Table 3:** Distribution of selected CAZy families biomass-degrading metagenomes.

		Proportion of GH detected (%)					
CAZy function	GH family*	55°C reactor 1	40°C reactor 1	Termite hindgut ^2^	Wallaby foregut ^3^	Compost ^4^	Cow rumen ^5^
Cellulases	GH5	3.12	2.27	7.96	1.80	2.58	5.23
	GH6	0	0	0	0	0.94	0
	GH7	0	0	0	0	0.17	> 0.01
	GH9	1.22	1.03	1.28	0	2.92	2.86
	GH44	0.08	0.03	0.85	0	0	*nr* 6
	GH45	0	0	0.57	0	0.09	0.41
	GH48*	1.07	0.27	0	0	0.09	0.01
Endo-	GH8*	0.15	0.77	0.71	0.18	0.69	1.19
hemicellulases	GH10*	3.65	1.95	6.54	1.97	4.21	3.69
	GH11	0.46	0.27	1.99	0	0.60	0.59
	GH12*	0	0	0	0	0.34	0
	GH26*	1.60	0.53	2.13	0.90	1.63	1.33
	GH28	1.07	1.51	0.85	0.36	0.77	1.70
	GH53	1.29	0.89	1.71	1.62	0.26	*nr*
Debranching	GH51*	3.96	2.16	2.56	2.15	1.03	*nr*
enzymes	GH54	0	0	0	0	0	*nr*
	GH62	0	0	0	0	0.43	> 0.01
	GH67	0.91	1.06	1.42	0.90	2.06	0.43
	GH78*	1.06	2.16	0	4.48	4.72	4.54
Oligosaccharide	GH1	8.90	8.80	3.13	10.95	6.78	0.91
degradation	GH2	4.41	5.23	3.27	4.31	3.78	5.17
	GH3	7.31	8.18	9.81	12.92	7.04	10.25
	GH29	1.90	1.59	0	0.36	2.23	3.38
	GH35	0.84	1.00	0.43	0.54	0.52	0.57
	GH38	1.98	2.04	1.56	0.54	1.55	0.98
	GH39*	2.13	1.30	0.43	0.18	0.94	1.13
	GH42*	1.98	2.92	3.41	1.44	1.89	1.34
	GH43*	5.78	8.68	2.28	1.80	7.81	*nr*
	GH52*	0.53	0.18	0.43	0	0	*nr*
All other GH		44.60	45.18	46.68	52.60	43.93	54.29
Total GH		1314	3387	703	557	1165	27,755

1 This study; ^2^ Warneck *et* *al*. 2007; ^3^ Pope *et* *al*. 2010; ^4^ Allgaier *et* *al*. 2010; ^5^ Hess *et* *al*. 2011.

6 *nr* – Not reported by authors.

* GH family names followed by an asterisk(*) indicate significant differences in GH family abundance between the reactor metagenomes, as detected by normalized log-odds ratios and false discovery rate correction.

**Figure 3:**
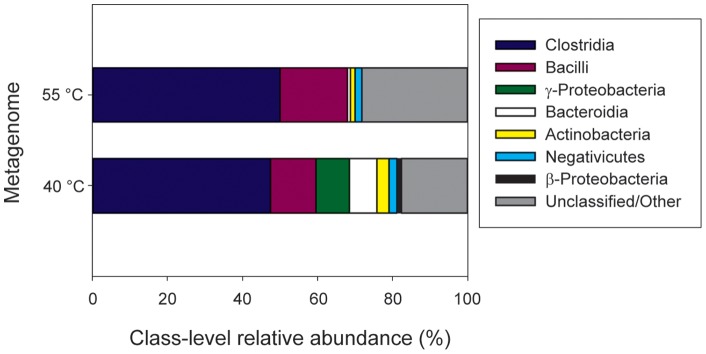
Phylogenetic distribution of metagenome reads according to best BLAST hits IMG database isolate genomes.

The phylogenetic distribution of sequence reads indicated that both fermentor metagenomes were dominated by genomes resembling *Clostridium*- and *Bacillus*-like isolates (Figure 3). In addition to these, reads associated with isolate genomes from the Bacteroidia, γ-Proteobacteria, β-Proteobacteria, and Actinobacteria were also detected. The two metagenomes also displayed a high degree of coverage for several isolate genomes from the bacterial classes mentioned above (Table 4). For example, the 55°C community contained sequence data representing approximately 89% of the protein coding sequences harbored by *Thermoanaerobaterium thermosaccharolyticum* DSM 571 and 86% of the protein coding sequences contained within *Symbiobacterium thermophilum* IAM 14863. Likewise, the 40°C community harbored genes for multiple nearly complete *Clostridium* spp. genomes, much of a genome resembling *Klebsiella pneumoniae*, and a large portion of a *Bacteroides* sp. genome. Protein recruitment plots of the metagenomes relative to these isolates are presented in Figures S1 and S2.

**Table 4:** Metagenome coverage of isolate genomes.^1^

Fermentation	Isolate genome	Metagenome homologs	Isolate genome protein coding genes 2	Coverage (%)
55°C	*Thermoanaerobacterium thermosaccharolyticum* DSM 571	2475	2770	89
	*Symbiobacterium thermophilum* IAM 14863	2872	3337	86
	*Moorella thermoacetica* ATCC 39073	1960	2523	77
	*Clostridium thermocellum* 3	2391	2979 – 3236	74 – 80
	*Geobacillus* sp.	3174	3477 – 3828	83 – 91
40°C	*Clostridium botulinum*	3872	2207 – 4097	94 – 175
	*Klebsiella pneumoniae*	4426	5195 – 5768	77 – 85
	*Clostridium saccharolyticum* DSM 2544	3040	4299	71
	*Bacteroides* sp.	3482	3652 – 5959	58 – 95
	*Clostridium* sp.	2492	2949 – 4266	58 – 86
	*Clostridium phytofermentans* ISDg	2301	3902	59

1 Ranges provided for isolate genome protein coding gene counts and coverage values are in cases where genome sequences of multiple isolates exist for a given species.

2 Values obtained from the IMG/M database (http://img.jgi.doe.gov/cgi-bin/m/main.cgi).

3 Isolate strain designations used in instances where ranges are provided include:

*C. thermocellum* (DSM 4150, DSM 2360).

*Geobacillus* sp. (C56-T3, WCH70, G11MC16, Y4.1MC1, Y12MC52).

*C. botulinum* (type A-Hall, BoNT/A1 ATCC 19397, BoNT/A1 Hall, F Langeland, BonT/A3 Loch Maree, B Eklund 17B, BoNT/B1 Okra, NCTC 2916, type C-Eklund, Bf, E3 Alaska E43, A2 Kyoto-F, Ba4 657, E1 BoNT Beluga, D 1873, F 230613 ).

*K. pneumoniae* (MGH78578, 342, NTUH-K2044, rhinoscleromatis ATCC 13884).

*Bacteroides* sp. (2_1_7, D2, dorei 5_1_36/D4, 9_1_42FAA, D1, 2_2_4, 1_1_6, 3_2_5, 4_3_47FAA, 2_1_16, 2_1_22, 2_1_33B, 3_1_33FAA, D20, 1_1_14, 20_3, 3_1_19, 3_1_23, D22).

*Clostridium* sp. ( L2-50, SS2/1, M62/1, 7_2 _3FAA).

Multiple metagenome sequence bins were parsed from the thermophilic and mesophilic fermentor communities (Table 5). Three major bins were identified within the 40°C metagenome and an additional 9 bins were identified within the 55°C metagenome. The bins from the mesophilic community corresponded to two organisms from the Bacteroidales and a member of the Actinomycetales; however, no bins resembling members of the Clostridia or Gammaproteobacteria (e.g., *Klebsiella*) were parsed successfully from the mesophilic sequence library. Bins generated from the thermophilic community were found to represent multiple members of the Clostridiales and Thermoanaerobacterales, as well as a member of the Bacillales. Single copy gene analysis was used to estimate bin completeness, and the identification of duplicate conserved single copy genes was used as an indicator of over-binning. Bin completeness ranged from 71–100%, but in some cases it appears that over-recruitment of sequence reads is likely to have occurred. In particular, Bin 10 from the thermophilic metagenome appears to represent multiple species or strains of *Thermoanaerobacterium*, and Bin 1 from the mesophilic metagenome is likely to represent multiple *Bacteroides*.

**Table 5:** Evaluation and functional characterization of metagenome contig bins.

Bin ID	Phylogenetic order	Sequence (kb)	Read depth	G+C (%)	Estimated completeness (%) 1	Duplicated CSCG (%) 2	Fermentation-related functions and features 3
55°C Bin 1	Clostridiales	1736	20.8	49	81.30	0.00	1, 2, 3, 5, 6, 7, 8
55°C Bin 2	Thermoanaerobacterales	846	10.1	43	95.34	11.86	1, 2, 3, 4, 5, 7
55°C Bin 3	Clostridiales	148	11.0	38	73.17	4.16	3, 5, 6, 8
55°C Bin 6	Thermoanaerobacterales	5545	18.7	35	95.34	63.63	1, 2, 3, 5, 6, 7, 8, 9, 10
55°C Bin 7/8	Clostridiales	2898	13.3	43	71.54	0.00	1, 2, 3, 5, 6, 7, 8, 9, 10
55°C Bin 10	Thermoanaerobacterales	2656	8.6	46	72.30	11.86	1, 2, 3, 5, 6, 7, 8, 9
55°C Bin 11	Bacillales	1980	10.8	49	71.92	11.86	1, 5, 6, 7, 8, 10
55°C Bin 12	Thermoanaerobacterales	2313	17.5	44	81.37	11.86	1, 2, 3, 5, 6, 7, 8
55°C Bin 13	Clostridiales	2307	12.1	40	72.36	7.69	1, 2, 3, 4, 6, 8
40°C Bin 1	Bacteroidales	4547	11.2	41	95.37	57.85	1, 3, 4, 6, 7, 8, 10
40°C Bin 5	Bacteroidales	3950	35.3	54	100	2.38	1, 2, 3, 4, 7, 8, 9
40°C Bin 1247	Actinomycetales	2458	10.7	57	95.47	10.71	2, 4, 5, 6

1 Estimated completeness  =  proportion of core genes relative to the total number of core genes expected in the order-level pangenome.

2 Duplicated conserved single copy genes (CSCG)  =  ratio of duplicated CSCG identified relative to the number of CSCGA detected.

3 Potential function inferred by pathway reconstruction using sequence matches to KEGG orthology terms, the presence of substrate- transport/uptake systems, and phenotype descriptions from the IMG/M system.1) Cellulose degradation; 2) Cellobiose uptake and/or degradation; 3) Xylan/xylose uptake and/or degradation; 4) Arabinose uptake and/or degradation; 5) “Multiple sugar" transporters (including maltose, mannose, and other simple sugars); 6) Acetate production; 7) Ethanol production; 8) Propanoate production; 9) Butanoate production; 10) Potential valerate/caproate production.

Major fermentation-related functions associated with each bin, as inferred through pathway reconstruction, are also presented in Table 5. Most bins appeared to have the potential to degrade cellulose, cellobiose, or xylose, as well as a variety of simple sugars. Likewise, the potential to produce acetate, ethanol, and propionate was distributed widely across the bins, but the potential for butanoate and caproate production was detected less frequently and limited to fewer sequence bins.

## Discussion

Limited understanding of the microbial ecology of the carboxylate platform has been identified as one of the major barriers to its adoption and implementation at large, industrially relevant scales [4]. It is known that an important interplay exists with respect to the physiology of the platform's microbial communities and the conditions under which they operate, but research regarding the ecology of these communities and the potential to manage their biomass conversion abilities is still in its early stages. Recent studies have begun to establish a baseline understanding of the types of organisms associated with the platform and the ways in which they vary under different operating conditions [9], [12], [13]. The results of the work described here extend these findings beyond 16S rRNA gene characterizations and provide new information regarding the metabolic potential harbored by platform bacteria.

The taxonomic composition of the fermentor metagenomes closely mirrors that which was observed using 16S rRNA gene pyrotag sequence libraries [9]. The thermophilic metagenome contained large numbers of sequences originating from *Thermoanaerobacterium*, Clostridia, and Bacilli, the same major taxa identified in the fermentor via 16S rRNA gene libraries. Likewise the mesophilic metagenome contained large numbers of sequence reads originating from the dominant members of its associated16S rRNA gene libraries, including members of the Clostridia, Bacteroidia, Proteobacteria, and Actinobacteria. In some cases, near-full length coverage was achieved for isolate genomes representing these taxa (Table 4).

Despite harboring communities that differed dramatically from a taxonomic point of view, the two metagenomes were quite similar to one another with respect to their functional gene content. Depending on the metric used (i.e., COG categories, Pfams, EC categories), 80 to 97% of functions were present in similar proportions across the two communities. As might be expected, many of these functions were related to central metabolism and general housekeeping, but they also included genes and pathways related to lignocellulose degradation. The two metagenomes shared similar types and abundances of cellulase (Table 3), but at finer levels of detail differences among genes related to substrate uptake and utilization were identified, complementing the variation observed between the fermentor metagenomes with respect to acid production and community composition.

Relative to the thermophilic metagenome, the mesophilic metagenome was significantly enriched in genes related to the degradation of hemicellulose-derived oligosaccharides, and more specifically, the five-carbon sugar, arabinose. In fact, nearly 9% of the glycosyl hydrolases identified in the mesophilic metagenome were related to GH43, a CAZy family composed of arabinases. The potential for enhanced metabolism of arabinose in the mesophilic metagenome makes sense given that the mesophilic community was dominated by Bacteroidete-like organisms. Many Bacteroidetes are known degraders of arabinose and other hemicellulose-derived sugars, and some Bacteroides sp. have the ability to convert arabinose to propionate [18]. Although we did not quantify arabinose concentrations in our fermentor system, we did quantify propionic acid concentrations (i.e., the conjugate acid to propionate). Propionic acid concentrations were significantly greater under mesophilic fermentation conditions (Table 1), and the combination of abundant Bacteroidetes and enriched arabinases provides a plausible explanation for enhanced propionic acid production.

In contrast to the mesophilic arabinase enrichment, the thermophilic metagenome was significantly enriched in genes related to the uptake of cellobiose. Although one might interpret this result to mean that the thermophilic metagenome had the potential to utilize cellobiose more effectively, we would suggest that the two communities were equipped to process cellobiose differently. In the thermophilic community, a *C. thermocellum*-like organism would be expected to degrade cellulose via (extracellular) cellulosomes [19], resulting in the release of cellobiose into the surrounding medium and creating a potential need for cellobiose transporters within the cellulose-degrader and among other members of the community. Indeed, many of the thermophilic taxa identified via taxonomic binning and isolate genome mapping efforts were equipped for the uptake and utilization of cellobiose. In contrast, the relative depletion of cellobiose transporters, coupled with the relative enrichment of glucosidases (Tables S4 and S5), in the mesophilic community suggests that extracellular degradation may be the dominant mode of cellobiose utilization when the platform is operated under mesophilic conditions.

In addition to differences related to substrate uptake and utilization, we also found the thermophilic metagenome to be significantly enriched in genes related to the transfer of genetic information, including transposases, viral integrases, and pilus proteins (Figure 2). Although the larger implications of this finding are uncertain, it is possible that the temperature conditions or limited diversity associated with the thermophilic community might be conducive to horizontal gene transfer [20]. Alternatively, the detection of these genes may be a function of the evolutionary history of the taxa we encountered, as horizontal gene transfer is believed to have played an important role in the development and distribution of cellulase systems [21].

Among the cellulose-degrading metagenomes described to date, it has been typical to find cellulases and hemicellulases accounting for 0.5% or more of protein-coding genes (e.g., [16], [17], [22]). Similarly, 0.45% of the protein-coding genes identified in the thermophilic metagenome, and 0.6% of the protein-coding genes identified in the mesophilic metagenome, fell into these categories. The carboxylate platform metagenomes also tended to resemble other lignocellulose-degrading metagenomes with respect to their general distribution of genes across glycosyl hydrolase families (Table 3). Two notable exceptions were the enrichment of GH48 (a family of cellobiohydrolases) and the depletion of GH78 (an α-L-rhamnosidase) in the carboxylate platform metagenomes relative to the compost, cow rumen, and Tamar wallaby metagenomes. Such shifts in GH abundance may be related to differences in community composition, feedstock composition (i.e., sorghum vs. switchgrass vs. mixed plant biomass), or the chemistry of the host environment. Given that these same GH families differed significantly between the two fermentor metagenomes (which utilized the same sorghum feedstock), community composition seems to be the most likely explanation.

In contrast to most of these other systems, our interest in the carboxylate platform communities extended beyond lignocellulose degradation and included the production of volatile fatty acids. Acetate, propionate, and n-butyrate typically dominate the product profile of the carboxylate platform, but smaller fractions of valerate, caproate, and heptanoate are also commonly produced [11]. Acetate typically accounts for >50% of the platform's product spectrum but may be produced in greater proportions under thermophilic conditions [7], [8]. The production of propionate and butyrate also tend to vary with temperature [9]. Propionate production is typically reduced under thermophilic conditions and was significantly so here (Table 1). In contrast, butyrate production tends to be enhanced under thermophilic conditions. Genes associated with the production of ethanol, acetate, propionate, and butyrate were found in both metagenomes, and pathway reconstruction efforts suggest the presence of full metabolic pathways for these products within many of the thermophilic and mesophilic metagenome bins (Table 4).

Although several of the thermophilic metagenome bins appear to have the ability to produce propionate, very little was detected in the thermophilic product pool following 16 days' fermentation. Closer inspection of the thermophilic metagenome indicates that in addition to possessing the suite of genes necessary for propanoate production, it also contains the genes necessary to perform propionate oxidation via the methylmalonyl-CoA pathway [23]. Through this pathway, propionate may be oxidized to acetate or butyrate. Thus, the lack of propionate in the thermophilic product pool may be the result of its utilization in the production of secondary metabolites. Alternatively, the propanoate pathway may not be utilized actively, but rather may be present as an adaptive strategy reserved for coping with changing environmental conditions or substrate availability.

Long-chain fatty acids, including valerate and caproate, were also of particular interest for this study, because of their high energy densities, the relative ease with which they can be converted into drop-in ready fuels, and their inherent coupling to H_2_ production [24], [25]. Valerate and caproate are typically produced through the secondary fermentation of ethanol or hydrogen and shorter-chain VFAs, in the absence of methanogens [25] (Figure 1B). The enzymes butyryl-CoA dehydrogenase and NADH: ferredoxin oxidoreductase (rnfABCDEFG) are considered key to the chain-elongation reactions that transform acetate to butyrate and butyrate to caproate [26], and a similar mechanism is thought to be responsible for the elongation of propionate to valerate [25]. Butyryl-coA dehydrogenase and acyl-coA dehydrogenases potentially involved in the production of longer-chain fatty acids were detected in both metagenomes. The COG category representing this group of genes (COG1960) was significantly enriched in the mesophilic metagenome (Table S4) and may have contributed to the enhanced production of valerate and caproate observed in the mesophilic fermentors.

Based parallel detection of functional genes and metabolic pathways within the fermentor metagenomes and across the metagenome sequence bins, the results of this study suggest that both metabolic flexibility (in terms of the types of substrates that may be metabolized) and a high level of functional redundancy are likely to be important to the carboxylate platform's ability to process lignocellulosic substrates. Although many cellulolytic microorganisms are considered to be specialists with respect to substrate preference and utilization [21], metabolic pathway reconstruction efforts focused within the fermentor metagenome sequence bins suggest that many of organisms identified were not limited to roles as specialist consumers, but rather, appear to have the ability to utilize a wide variety of cellulosic- and hemicellulosic-sugars. Likewise, many of these organisms also appear to share the potential to produce multiple fermentation products, including acetate and/or ethanol, propionate, and butanoate. The presence of parallel metabolic pathways within each of the fermentor communities may confer a degree of stability to the fermentation process [27], despite evidence suggesting that the composition of the communities themselves may be flexible and dynamic [9].

Historically, mixed-community fermentations have been perceived as unstable and unpredictable [4], [28]. As sequencing technologies open the door to larger-scale and longer-term characterization of these communities, new evidence is emerging to suggest that these systems are more predictable than previously thought [29]. It is anticipated that coupling an understanding of the functional potential of fermentor communities, such as those described here, with studies that evaluate the range of community responses to perturbation and changes in operating parameters will be invaluable to our ability to control and predict fermentor performance and move forward in the implementation of these technologies at large, industrially relevant scales.

## Materials and Methods

### Feedstock preparation, inoculum source, and fermentor construction

As described in Hollister et al. [9], biomass from a photo-period sensitive, high-tonnage sorghum cultivar (*Sorghum bicolor* (L.) Moench) was obtained from the Sorghum Breeding and Genetics Program at Texas A&M University and used as feedstock. Prior to its use, the sorghum was dried, chipped, and treated with hot water and lime (0.1 g Ca(OH)_ 2_) and 10 mL distilled H_2_O per g dry biomass; 2 h at 100°C) to enhance its digestibility [30].

Marine sediment, collected from Galveston, TX, USA, has proven to be one of the best-performing carboxylate platform inoculum sources identified to date [10]. As such, sediment collected from Galveston served as the reactor inoculum. Sediment was collected from a series of shoreline pits, at a depth of 0.5 m, the point at which the sediment's color transitioned from yellow/brown to dark gray/black. Sediment samples were placed into bottles containing deoxygenated water, 0.275 g L^−1^ sodium sulfate, and 0.275 g L^−1^ cysteine hydrochloride, as described by Thanakoses *et* *al.* [10]. The bottles were held on ice during transport to the laboratory, and then they were stored at −20°C until later use. Prior to inoculation, a single sediment sample was thawed, shaken vigorously, and allowed to settle by gravity. Aliquots of the resulting supernatant were used to inoculate the fermentor vessels.

Fermentations were performed in a series of 1-L polypropylene centrifuge bottles fitted with a stirring and venting apparatus [8]. Each fermentor contained 50 mL marine sediment inoculum, 36 g lime-treated sorghum, 4 g dried chicken manure (included as a nutrient source and potential source of additional inoculum; obtained from the Poultry Science Center at Texas A&M University, College Station, TX), and 350 mL deoxygenated water, as well as calcium carbonate buffer (CaCO_3_, 15 g L^−1^) and iodoform (CH_3_I, 20 g L^−1^, used to inhibit methane production). Fermentors were flushed with N_2_ prior to capping and were rolled continuously at 2 rpm throughout their incubation. Two incubation temperatures (40 and 55°C) were utilized, and the fermentors were set up in such a way that a set (*n* = 3) of vessels from each temperature treatment could be sacrificed for DNA extraction. The metagenomes described here were collected as a part of a larger study aimed at characterizing carboxylate platform microbial community dynamics at multiple time points in a typical laboratory-scale fermentation [9].

### Fermentor monitoring and sample collection

Carbon dioxide (CO_2_) and methane (CH_4_) production, pH, and total carboxylic acid concentrations were monitored every two days over the course of the incubation, and as fermentations were terminated, samples of both the solid and liquid phases were collected for chemical analysis. Fermentor vessels were centrifuged in a Beckman J-6B centrifuge (Beckman Coulter, Inc., Brea, CA, USA) with a swinging bucket rotor at 3297×*g* for 30 minutes to separate fermentor solids and liquids. An aliquot of supernatant was collected and subjected to carboxylic acid analysis, as described by Hollister et al. [9], and solids were analyzed to determine the mass of the remaining undigested volatile solids (VS). The solids were first dried at 105°C and then ashed at 550°C [8]. The VS content of each sample was calculated as the difference between its oven dry weight and its ashed weight.

Fermentor performance was characterized at multiple time points using metrics such as conversion, selectivity, yield, and productivity. Conversion was quantified as the proportion of VS that had been digested relative to the quantity of VS initially loaded into the fermentor. Selectivity was calculated as the fraction of digested material converted specifically to carboxylic acids. Yield was determined by calculating the ratio of total carboxylic acids produced relative to the quantity of VS initially loaded into the reactor, and productivity was defined as the rate of acid production (g acid L^−1^ d ^−1^). Comparisons of these values, as well as the relative abundances of various acid products at the mid-point of the fermentation (i.e., when the metagenome samples were collected), were conducted using paired, two-tailed Student's t-tests, and *p*-values <0.05 were considered to represent significant differences.

### DNA extraction

Fermentor materials for the shotgun metagenome sequence libraries were collected after 16 days' incubation, the approximate mid-point and typically most productive stage for laboratory-scale carboxylate platform batch fermentations. Solids and liquids from each replicate were combined in equal volumes to create a single composite sample for each temperature condition. The composites were stored at −80°C until DNA extraction. Just prior to extraction, fermentor samples were thawed and centrifuged at 4000×*g* for 10 min. DNA was extracted from the pellet materials using a PowerMax soil DNA extraction kit (Mo Bio Laboratories, Inc., Carlsbad, CA, USA), using a lysozyme-modified version of the manufacturer's protocol [31]. Following elution, DNA samples were concentrated via ethanol precipitation and purified using illustra MicroSpin S-400 HR columns (GE Healthcare Bio-Sciences Corp, Piscataway, NJ, USA). DNA samples were quality checked according to US DOE Joint Genome Institute (JGI) protocols (http://my.jgi.doe.gov/general/index.html) and were submitted to the JGI for sequencing.

### Metagenome sequencing, assembly and analysis

DNA from the fermentor samples was used to construct 454 standard shotgun sequencing libraries according to manufacturer's recommended protocols. An additional 8 kb insert paired-end 454 library was constructed from the 40°C fermentor DNA. A total of two full runs of 454 Titanium sequencing were completed for each of the two communities: one shotgun and one paired-end for the 40°C community, and one run from each of two shotgun libraries for the 55°C community. This yielded a total of 588 Mb (∼2.58 million reads) and 900 Mb (∼2.59 million reads) of raw sequence for the 40°C and 55°C communities, respectively.

Sequence reads were quality trimmed to an accuracy of 99.3% using LUCY [32] and duplicate reads were identified and removed prior to assembly. Filtered and quality trimmed reads were assembled with Newbler version 2.4. Approximately 67% of the filtered reads from the 40°C sample and 92% of the filtered reads from the 55°C sample assembled into contigs, which represented 58% and 76% of raw reads, respectively. All resulting contigs and unassembled singlet reads were submitted to IMG/M [33], a metagenome-specific version of the Integrated Microbial Genomes (IMG) database annotation pipeline [34], which includes multiple gene-finding algorithms and BLASTx search capabilities. Reads were annotated through comparison with the KEGG database via BLASTx, using an e-value cutoff of 1×10^−5^ [34], and enzyme EC numbers were assigned based upon KEGG orthology (KO) terms [33]. COGs were identified via a reverse PSI-BLAST of the CDD database, using an e-value cutoff of 1×10^−2^ [34]. The phylogenetic distribution of the metagenome protein coding sequences was determined using best BLASTp hits to sequenced isolate genomes at similarity cutoffs ranging from 30 to 90% [33]. Coverage of these isolate genomes was determined as described by Lykidis et al. [35], whereby the proportion of best-BLAST hits to metagenome protein coding genes was calculated relative to the total number of protein coding genes contained in each isolate genome. Differences in gene content (e.g., COGs, enzyme categories, or Pfam classes) were identified using a Z-normalized log odds ratio test, which evaluated the relative enrichment or underrepresentation of gene categories between the two metagenomes. Significance values were adjusted for multiple comparisons using a false discovery rate correction equivalent to *p*<0.05. Specific corrected *p*-value cutoffs for KEGG, COG, and enzyme category comparisons are provided in Tables S3, S4, and S5, respectively.

Searches for glycosyl hydrolases (GH), as identified by the CAZy database [36] and described by Warnecke et al. [16], were performed through BLASTx searches and by evaluating hits to Pfam hidden Markov models (HMM) in the IMG/M system. Top hits to each contig were utilized. An e-value cutoff of 10^−6^ was used in conjunction with our BLAST results, and HMM searches were implemented as described in Mavromatis et al. [34]. Differences in GH abundance were evaluated using the Z-normalized log odds ratio test as described above, and *p*-values were adjusted for multiple comparisons using a false discovery rate correction equivalent to *p*<0.05.

The Classifier for Metagenomic Sequences software tool (ClaMS-CLI; http://clams.jgi-psf.org/) was used to cluster the metagenomic sequences into phylogenetic bins. The binning of metagenomic sequences attempts to separate sequence data into clusters that represent the taxa from which they were originally derived. A kmer length of 3 was used in conjunction with a de Bruijn chain algorithm, a distance cut-off of 0.01, and a training set constructed from phylogenetic marker COGs that were identified within each metagenome using IMG/M. Potential outlier sequences were removed from bins on the basis of G+C content (%) and depth of coverage; those that deviated more than one standard deviation of the mean for G+C (%) and/or depth of coverage from their respective bins were excluded from further analyses.

Bin completeness was evaluated using pangenomic and single-copy gene approaches, as described by Hess et al. [14]. Best BLAST hits of the protein coding genes contained within each metagenome bin were used to assign identities at the phylgenetic order level. Collections of COGs from genomes corresponding to the order of each bin were assembled from the finished genomes available in the IMG database [32]. Those COGs that appeared in all genomes of a given order were designated as core to the pangenome and were used as the basis for evaluating bin completeness (i.e. the % of core genes identified). Single copy genes that occurred in a conserved manner across all available finished genomes at a given phylogentic order were used to evaluate potential “over-binning” among the sequence bins, whereby the number of conserved single copy genes that were detected multiple times were expressed as a proportion of the total number of single copy genes expected. The identities of the genomes used are provided in Table S6. Following bin verifications, the functional pathways contained within each bin were reconstructed utilizing KEGG orthology terms and the MinPath software package [37].

Metagenome sequence data are available through the IMG/M system (http://img.jgi.doe.gov/m) and are identified as “Mixed Alcohol (MixAlco) bioreactor” samples. Sequence data may also be accessed through the NCBI Sequence Read Archive under accession SRA044949.

## Supporting Information

Figure S1 **Protein recruitment plots of the thermophilic metagenome versus high-coverage isolate genomes.** The length of each genome is depicted along the *x*-axis. BLAST hits with >30% identity are indicated by blue, hits with >60% identity are indicated by green, and hits with >90% identity are indicated by red.
(TIF)

Figure S2 **Protein recruitment plots of the mesophilic metagenome versus high-coverage isolate genomes.** The length of each genome is depicted along the *x*-axis. BLAST hits with >30% identity are indicated by blue, hits with >60% identity are indicated by green, and hits with >90% identity are indicated by red.
(TIF)

Table S1 **Fermentor performance metrics following 16 days**' **incubation.**
(DOC)

Table S2 **Proportion of protein coding genes (%) receiving a functional annotation within each of the databases listed.**
(DOC)

Table S3 **Pfams significantly enriched or depleted between the thermophilic and mesophilic metagenomes, as determined using a** ***z*** **-normalized log odds ratios (Z-LOR).**
(DOC)

Table S4 **COGs significantly enriched or depleted between the thermophilic and mesophilic metagenomes, as determined by** ***z*** **-normalized log odds ratios (Z-LOR).**
(DOC)

Table S5 **Enzymes significantly enriched or depleted between the thermophilic and mesophilic metagenomes, as determined by** ***z*** **-normalized log odds ratios (Z-LOR).**
(DOC)

Table S6 **Genomes used to identify phylogenetic order-level core genes and conserved single copy genes (CSGS).**
(DOC)
