# Supplementary figures and images for: Mesophilic and Thermophilic Conditions Select for Unique but Highly Parallel Microbial Communities to Perform Carboxylate Platform Biomass Conversion
Source: PLoS One. 2012 Jun 22;7(6):e39689. doi: 10.1371/journal.pone.0039689 (PMC3382152; doi:10.1371/journal.pone.0039689)

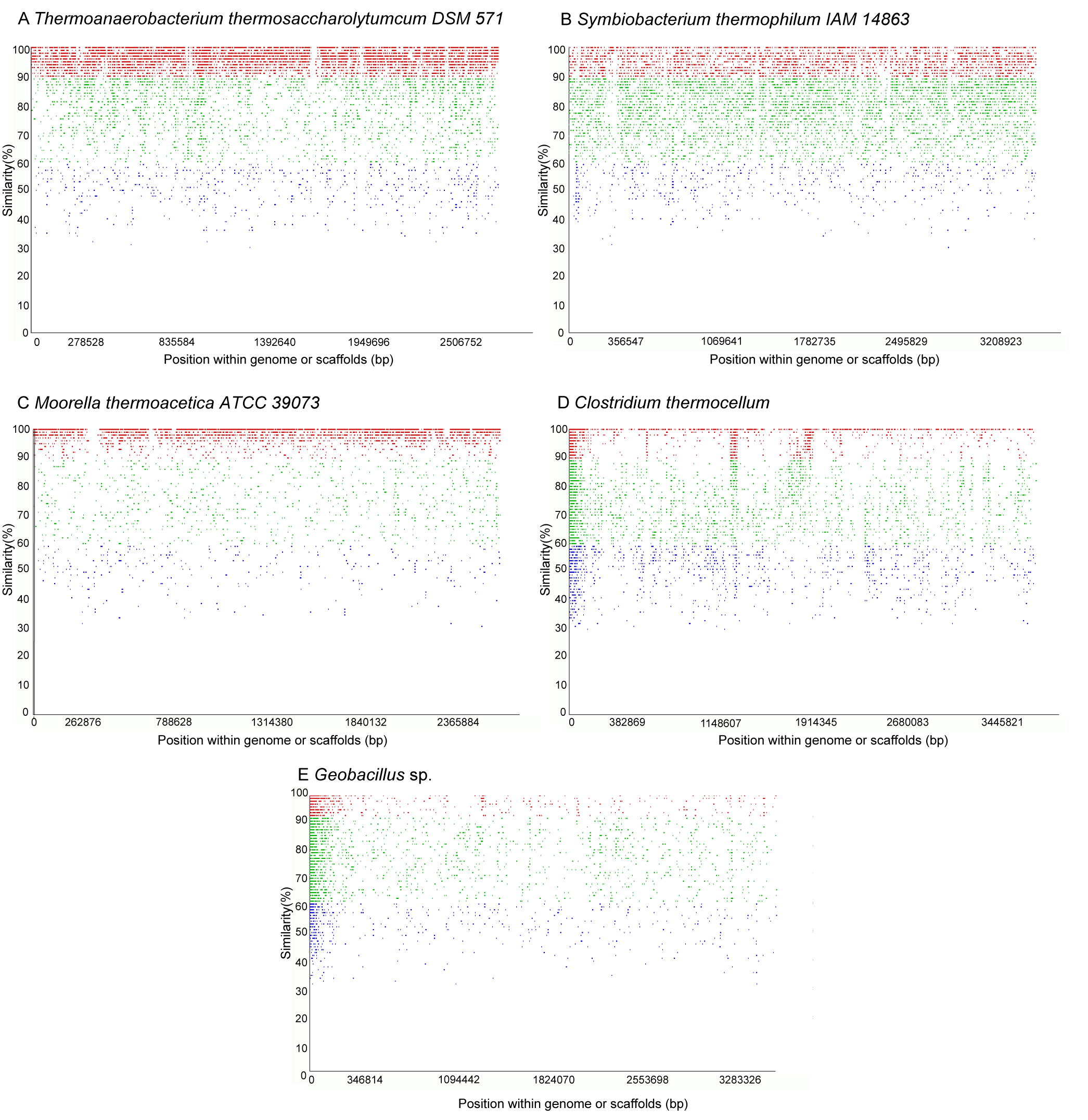

Supplement: Figure S1 — Protein recruitment plots of the thermophilic metagenome versus high-coverage isolate genomes. The length of each genome is depicted along the x-axis. BLAST hits with >30% identity are indicated by blue, hits with >60% identity are indicated by green, and hits with >90% identity are indicated by red. (TIF) [file pone.0039689.s001.tif]

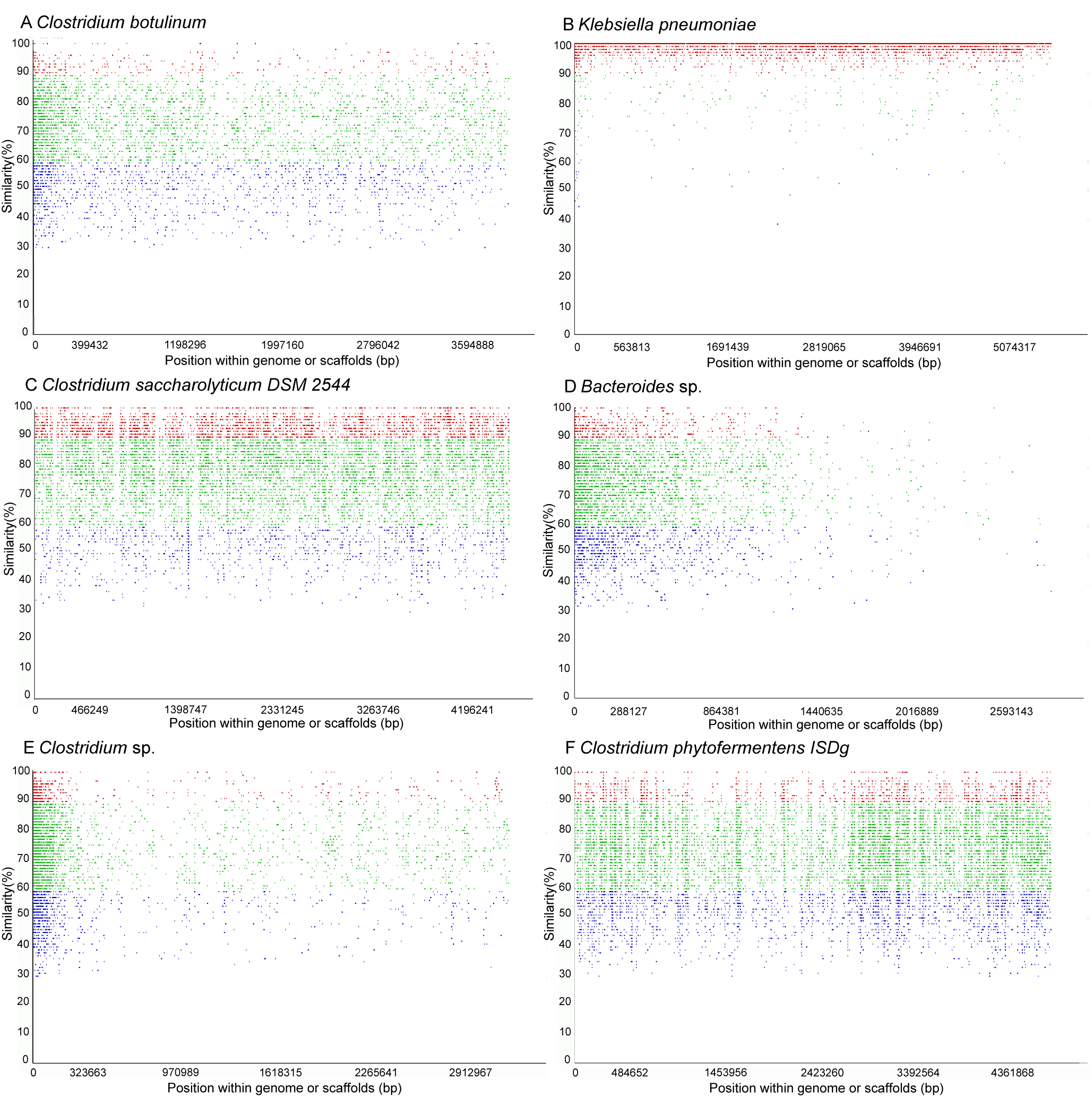

Supplement: Figure S2 — Protein recruitment plots of the mesophilic metagenome versus high-coverage isolate genomes. The length of each genome is depicted along the x-axis. BLAST hits with >30% identity are indicated by blue, hits with >60% identity are indicated by green, and hits with >90% identity are indicated by red. (TIF) [file pone.0039689.s002.tif]
