# Supplementary material for: Mesophilic and Thermophilic Conditions Select for Unique but Highly Parallel Microbial Communities to Perform Carboxylate Platform Biomass Conversion
Source: PLoS One. 2012 Jun 22;7(6):e39689. doi: 10.1371/journal.pone.0039689 (PMC3382152; doi:10.1371/journal.pone.0039689)
Supplement: Table S1 — Fermentor performance metrics following 16 days' incubation. (DOC) [file pone.0039689.s003.doc]

**Table S1. Fermentor performance metrics following 16 days’ incubation.**

| Treatment | Conversion^1^ | Selectivity ^2^ | Yield ^3^ | Productivity  (g acid L^−1^ d^−1^) |
| --- | --- | --- | --- | --- |
| 40 °C fermentation | 0.29 ± 0.01 | 0.31 ± 0.02 | 0.09 ± 0.01 | 0.46 ± 0.03 |
| 55 °C fermentation | 0.31 ± 0.02 | 0.24 ± 0.01 | 0.08 ± 0.02 | 0.39 ± 0.02 |

^1^ Conversion represents the ratio of volatile solids digested to the biomass that was originally loaded into the reactor.

^2^ Selectivity represents the proportion of digested material that resulted in carboxylic acid production.

^3^ Yield represents the ratio of total carboxylic acids produced to the biomass that was originally loaded into the reactor.
