# Supplementary material for: Mesophilic and Thermophilic Conditions Select for Unique but Highly Parallel Microbial Communities to Perform Carboxylate Platform Biomass Conversion
Source: PLoS One. 2012 Jun 22;7(6):e39689. doi: 10.1371/journal.pone.0039689 (PMC3382152; doi:10.1371/journal.pone.0039689)
Supplement: Table S2 — Proportion of protein coding genes (%) receiving a functional annotation within each of the databases listed. (DOC) [file pone.0039689.s004.doc]

**Table S2.** Proportion of protein coding genes (%) receiving a functional annotation within each of the databases listed.

|  | Proportion of protein coding genes assigned a function (%) | | | | |
| --- | --- | --- | --- | --- | --- |
| Treatment | Having predicted function | KEGG pathways | KEGG orthology | COGs | Pfam |
| 40 °C fermentation | 60.74 | 27.27 | 45.04 | 48.77 | 47.54 |
| 55 °C fermentation | 57.11 | 26.62 | 45.09 | 48.39 | 49.07 |
