# Supplementary material for: Mesophilic and Thermophilic Conditions Select for Unique but Highly Parallel Microbial Communities to Perform Carboxylate Platform Biomass Conversion
Source: PLoS One. 2012 Jun 22;7(6):e39689. doi: 10.1371/journal.pone.0039689 (PMC3382152; doi:10.1371/journal.pone.0039689)
Supplement: Table S3 — Pfams significantly enriched or depleted between the thermophilic and mesophilic metagenomes, as determined using a z -normalized log odds ratios (Z-LOR). (DOC) [file pone.0039689.s005.doc]

**Table S3.** Pfams significantly enriched or depleted between the thermophilic and mesophilic metagenomes, as determined using a *z*-normalized log odds ratios (Z-LOR). ^1^

| Pfam ID | Name | 40 °C gene count ^2^ | 55 °C gene count | Z-LOR | *p* value |
| --- | --- | --- | --- | --- | --- |
| pfam00395 | SLH | 129 | 277 | 12.37 | 0.00E+00 |
| pfam07833 | Cu_amine_oxidN1 | 59 | 171 | 10.72 | 0.00E+00 |
| pfam06782 | UPF0236 | 31 | 126 | 9.76 | 0.00E+00 |
| pfam01555 | N6_N4_Mtase | 96 | 166 | 8.59 | 0.00E+00 |
| pfam04055 | Radical_SAM | 339 | 359 | 8.08 | 3.33E-16 |
| pfam00872 | Transposase_mut | 154 | 202 | 7.72 | 6.00E-15 |
| pfam01385 | Transposase_2 | 28 | 81 | 7.37 | 8.38E-14 |
| pfam07282 | Transposase_35 | 26 | 75 | 7.09 | 6.76E-13 |
| pfam00665 | rve | 210 | 230 | 6.76 | 6.94E-12 |
| pfam01909 | NTP_transf_2 | 27 | 71 | 6.73 | 8.72E-12 |
| pfam04545 | Sigma70_r4 | 149 | 180 | 6.71 | 9.79E-12 |
| pfam01208 | URO-D | 33 | 73 | 6.43 | 6.58E-11 |
| pfam03845 | Spore_permease | 71 | 106 | 6.23 | 2.41E-10 |
| pfam01547 | SBP_bac_1 | 603 | 502 | 6.14 | 4.07E-10 |
| pfam02302 | PTS_IIB | 76 | 105 | 5.82 | 2.87E-09 |
| pfam05685 | DUF820 | 11 | 40 | 5.42 | 3.01E-08 |
| pfam01850 | PIN | 4 | 39 | 5.39 | 3.48E-08 |
| pfam04015 | DUF362 | 19 | 47 | 5.37 | 3.97E-08 |
| pfam00528 | BPD_transp_1 | 1657 | 1164 | 5.27 | 6.77E-08 |
| pfam01642 | MM_CoA_mutase | 36 | 62 | 5.24 | 8.15E-08 |
| pfam09643 | YopX | 8 | 34 | 5.09 | 1.78E-07 |
| pfam02511 | Thy1 | 16 | 41 | 5.07 | 1.98E-07 |
| pfam01934 | DUF86 | 12 | 37 | 5.06 | 2.14E-07 |
| pfam03787 | RAMPs | 1 | 93 | 5.06 | 2.10E-07 |
| pfam01261 | AP_endonuc_2 | 141 | 147 | 5.05 | 2.18E-07 |
| pfam04014 | SpoVT_AbrB | 36 | 60 | 5.05 | 2.21E-07 |
| pfam00676 | E1_dh | 46 | 69 | 5.04 | 2.33E-07 |
| pfam01833 | TIG | 5 | 30 | 4.86 | 6.00E-07 |
| pfam04542 | Sigma70_r2 | 364 | 305 | 4.86 | 5.89E-07 |
| pfam01881 | Cas_Cas6 | 6 | 30 | 4.84 | 6.59E-07 |
| pfam03069 | FmdA_AmdA | 6 | 29 | 4.75 | 1.03E-06 |
| pfam01844 | HNH | 48 | 67 | 4.69 | 1.35E-06 |
| pfam07670 | Gate | 103 | 112 | 4.67 | 1.50E-06 |
| pfam07697 | 7TMR-HDED | 9 | 30 | 4.62 | 1.88E-06 |
| pfam02310 | B12-binding | 66 | 81 | 4.57 | 2.39E-06 |
| pfam09704 | Cas_Cas5d | 14 | 34 | 4.54 | 2.84E-06 |
| pfam00534 | Glycos_transf_1 | 315 | 264 | 4.52 | 3.06E-06 |
| pfam02151 | UVR | 79 | 91 | 4.52 | 3.08E-06 |
| pfam11385 | DUF3189 | 4 | 26 | 4.52 | 3.15E-06 |
| pfam01420 | Methylase_S | 87 | 97 | 4.49 | 3.61E-06 |
| pfam03681 | UPF0150 | 7 | 27 | 4.49 | 3.59E-06 |
| pfam05709 | Sipho_tail | 33 | 51 | 4.43 | 4.78E-06 |
| pfam02604 | PhdYeFM | 12 | 31 | 4.42 | 4.93E-06 |
| pfam03061 | 4HBT | 61 | 75 | 4.41 | 5.17E-06 |

**Table S3.** continued.

| Pfam ID | Name | 40 °C gene count | 55 °C gene count | Z-LOR | *p* value |
| --- | --- | --- | --- | --- | --- |
| pfam05239 | PRC | 37 | 54 | 4.37 | 6.33E-06 |
| pfam04232 | SpoVS | 24 | 42 | 4.35 | 6.78E-06 |
| pfam00874 | PRD | 110 | 113 | 4.34 | 7.27E-06 |
| pfam04308 | DUF458 | 5 | 24 | 4.32 | 7.90E-06 |
| pfam04754 | Transposase_31 | 15 | 33 | 4.31 | 8.16E-06 |
| pfam04029 | 2-ph_phosp | 12 | 30 | 4.30 | 8.40E-06 |
| pfam09991 | DUF2232 | 12 | 30 | 4.30 | 8.40E-06 |
| pfam00460 | Flg_bb_rod | 99 | 104 | 4.29 | 8.74E-06 |
| pfam02255 | PTS_IIA | 30 | 47 | 4.29 | 8.91E-06 |
| pfam02754 | CCG | 29 | 46 | 4.28 | 9.31E-06 |
| pfam09484 | Cas_TM1802 | 1 | 41 | 4.22 | 1.24E-05 |
| pfam06947 | DUF1290 | 5 | 23 | 4.21 | 1.25E-05 |
| pfam02632 | BioY | 27 | 43 | 4.15 | 1.66E-05 |
| pfam02780 | Transketolase_C | 128 | 124 | 4.14 | 1.71E-05 |
| pfam09388 | SpoOE-like | 6 | 23 | 4.14 | 1.74E-05 |
| pfam05949 | DUF881 | 24 | 40 | 4.12 | 1.87E-05 |
| pfam07949 | YbbR | 24 | 40 | 4.12 | 1.87E-05 |
| pfam07195 | FliD_C | 33 | 48 | 4.11 | 2.02E-05 |
| pfam00269 | SASP | 53 | 65 | 4.09 | 2.11E-05 |
| pfam01558 | POR | 125 | 121 | 4.09 | 2.18E-05 |
| pfam01548 | Transposase_9 | 58 | 69 | 4.08 | 2.22E-05 |
| pfam02595 | Gly_kinase | 57 | 68 | 4.07 | 2.39E-05 |
| pfam03808 | Glyco_tran_WecB | 16 | 32 | 4.07 | 2.33E-05 |
| pfam05582 | Peptidase_U57 | 11 | 27 | 4.06 | 2.47E-05 |
| pfam05635 | Ribosomal_S23p | 10 | 26 | 4.06 | 2.50E-05 |
| pfam08761 | dUTPase_2 | 8 | 24 | 4.05 | 2.59E-05 |
| pfam07943 | PBP5_C | 43 | 56 | 4.03 | 2.75E-05 |
| pfam02811 | PHP | 170 | 153 | 4.02 | 2.86E-05 |
| pfam01368 | DHH | 79 | 85 | 4.01 | 3.01E-05 |
| pfam01966 | HD | 351 | 277 | 3.95 | 3.96E-05 |
| pfam01520 | Amidase_3 | 197 | 171 | 3.94 | 3.99E-05 |
| pfam09660 | DUF2397 | 10 | 25 | 3.93 | 4.27E-05 |
| pfam06429 | DUF1078 | 104 | 103 | 3.91 | 4.54E-05 |
| pfam03747 | ADP_ribosyl_GH | 53 | 63 | 3.90 | 4.85E-05 |
| pfam07501 | G5 | 53 | 63 | 3.90 | 4.85E-05 |
| pfam01314 | AFOR_C | 33 | 46 | 3.88 | 5.16E-05 |
| pfam03610 | EIIA-man | 32 | 45 | 3.87 | 5.47E-05 |
| pfam02730 | AFOR_N | 19 | 33 | 3.84 | 6.16E-05 |
| pfam03023 | MVIN | 43 | 54 | 3.82 | 6.58E-05 |
| pfam05504 | Spore_GerAC | 53 | 62 | 3.80 | 7.29E-05 |
| pfam01867 | Cas_Cas1 | 52 | 61 | 3.78 | 7.86E-05 |
| pfam05168 | HEPN | 6 | 20 | 3.78 | 7.97E-05 |
| pfam07352 | Phage_Mu_Gam | 2 | 19 | 3.77 | 8.06E-05 |
| pfam09021 | HutP | 5 | 19 | 3.76 | 8.58E-05 |
| pfam01930 | Cas_Cas4 | 23 | 36 | 3.75 | 8.75E-05 |
| pfam01040 | UbiA | 55 | 63 | 3.74 | 9.34E-05 |

**Table S3.** continued

| Pfam ID | Name | 40 °C gene count | 55 °C gene count | Z-LOR | *p* value |
| --- | --- | --- | --- | --- | --- |
| pfam00070 | Pyr_redox | 251 | 205 | 3.73 | 9.46E-05 |
| pfam01225 | Mur_ligase | 69 | 74 | 3.73 | 9.70E-05 |
| pfam05130 | FlgN | 21 | 34 | 3.73 | 9.54E-05 |
| pfam00480 | ROK | 146 | 131 | 3.70 | 1.07E-04 |
| pfam01169 | UPF0016 | 12 | 25 | 3.67 | 1.23E-04 |
| pfam06605 | DUF1142 | 12 | 25 | 3.67 | 1.23E-04 |
| pfam07285 | DUF1444 | 6 | 19 | 3.64 | 1.34E-04 |
| pfam00482 | GSPII_F | 94 | 92 | 3.63 | 1.42E-04 |
| pfam10079 | DUF2317 | 5 | 18 | 3.63 | 1.42E-04 |
| pfam00359 | PTS_EIIA_2 | 112 | 105 | 3.60 | 1.58E-04 |
| pfam03698 | UPF0180 | 2 | 17 | 3.60 | 1.57E-04 |
| pfam04230 | PS_pyruv_trans | 35 | 45 | 3.57 | 1.77E-04 |
| pfam04285 | DUF444 | 17 | 29 | 3.56 | 1.85E-04 |
| pfam01520 | Amidase_3 | 197 | 171 | 3.94 | 3.99E-05 |
| pfam02834 | 2_5_RNA_ligase | 14 | 26 | 3.54 | 2.02E-04 |
| pfam05913 | DUF871 | 33 | 43 | 3.54 | 2.03E-04 |
| pfam02491 | FtsA | 44 | 52 | 3.52 | 2.17E-04 |
| pfam09818 | ABC_ATPase | 8 | 20 | 3.51 | 2.21E-04 |
| pfam03793 | PASTA | 66 | 69 | 3.47 | 2.56E-04 |
| pfam00989 | PAS | 98 | 93 | 3.46 | 2.67E-04 |
| pfam01656 | CbiA | 261 | 207 | 3.46 | 2.70E-04 |
| pfam03144 | GTP_EFTU_D2 | 155 | 134 | 3.46 | 2.70E-04 |
| pfam06114 | DUF955 | 41 | 49 | 3.46 | 2.72E-04 |
| pfam06253 | MTTB | 1 | 20 | 3.46 | 2.66E-04 |
| pfam09861 | DUF2088 | 28 | 38 | 3.45 | 2.81E-04 |
| pfam04041 | DUF377 | 64 | 67 | 3.43 | 3.02E-04 |
| pfam01478 | Peptidase_A24 | 26 | 36 | 3.42 | 3.18E-04 |
| pfam07963 | N_methyl | 33 | 42 | 3.42 | 3.17E-04 |
| pfam01402 | RHH_1 | 16 | 27 | 3.41 | 3.21E-04 |
| pfam09648 | YycI | 2 | 15 | 3.41 | 3.22E-04 |
| pfam07929 | PRiA4_ORF3 | 15 | 26 | 3.40 | 3.32E-04 |
| pfam03646 | FlaG | 13 | 24 | 3.39 | 3.52E-04 |
| pfam05362 | Lon_C | 120 | 108 | 3.38 | 3.62E-04 |
| pfam03780 | DUF322 | 56 | 60 | 3.35 | 4.02E-04 |
| pfam08680 | DUF1779 | 5 | 16 | 3.35 | 4.03E-04 |
| pfam01695 | IstB | 156 | 133 | 3.34 | 4.18E-04 |
| pfam02585 | PIG-L | 21 | 31 | 3.34 | 4.24E-04 |
| pfam03961 | DUF342 | 51 | 56 | 3.34 | 4.13E-04 |
| pfam07155 | DUF1393 | 41 | 48 | 3.34 | 4.12E-04 |
| pfam05103 | DivIVA | 28 | 37 | 3.32 | 4.45E-04 |
| pfam07228 | SpoIIE | 50 | 55 | 3.32 | 4.48E-04 |
| pfam06792 | UPF0261 | 2 | 14 | 3.31 | 4.72E-04 |
| pfam07854 | DUF1646 | 2 | 14 | 3.31 | 4.72E-04 |
| pfam04411 | DUF524 | 3 | 14 | 3.29 | 4.98E-04 |
| pfam06941 | NT5C | 3 | 14 | 3.29 | 4.98E-04 |
| pfam10776 | DUF2600 | 3 | 14 | 3.29 | 4.98E-04 |

**Table S3.** continued

| Pfam ID | Name | 40 °C gene count | 55 °C gene count | Z-LOR | *p* value |
| --- | --- | --- | --- | --- | --- |
| pfam01274 | Malate_synthase | 16 | 26 | 3.27 | 5.37E-04 |
| pfam00115 | COX1 | 37 | 44 | 3.26 | 5.59E-04 |
| pfam00491 | Arginase | 61 | 63 | 3.26 | 5.53E-04 |
| pfam08281 | Sigma70_r4_2 | 204 | 165 | 3.26 | 5.52E-04 |
| pfam00595 | PDZ | 121 | 107 | 3.25 | 5.85E-04 |
| pfam01609 | Transposase_11 | 148 | 126 | 3.24 | 5.96E-04 |
| pfam01894 | UPF0047 | 13 | 23 | 3.24 | 5.98E-04 |
| pfam09548 | Spore_III_AB | 13 | 23 | 3.24 | 5.98E-04 |
| pfam12323 | HTH_14 | 13 | 23 | 3.24 | 5.98E-04 |
| pfam05448 | AXE1 | 23 | 32 | 3.23 | 6.12E-04 |
| pfam06032 | DUF917 | 12 | 22 | 3.23 | 6.15E-04 |
| pfam08721 | TnsA_C | 1 | 16 | 3.23 | 6.26E-04 |
| pfam09700 | Cas_Cmr3 | 1 | 16 | 3.23 | 6.26E-04 |
| pfam01476 | LysM | 248 | 194 | 3.22 | 6.50E-04 |
| pfam09581 | Spore_III_AF | 10 | 20 | 3.22 | 6.43E-04 |
| pfam01361 | Tautomerase | 6 | 16 | 3.21 | 6.75E-04 |
| pfam01944 | DUF95 | 7 | 17 | 3.21 | 6.67E-04 |
| pfam03323 | GerA | 223 | 177 | 3.21 | 6.72E-04 |
| pfam04471 | Mrr_cat | 9 | 19 | 3.21 | 6.53E-04 |
| pfam06271 | RDD | 6 | 16 | 3.21 | 6.75E-04 |
| pfam11104 | Competence_A | 8 | 18 | 3.21 | 6.61E-04 |
| pfam01996 | F420_ligase | 5 | 15 | 3.20 | 6.88E-04 |
| pfam07537 | CamS | 5 | 15 | 3.20 | 6.88E-04 |
| pfam01336 | tRNA_anti | 139 | 119 | 3.19 | 7.12E-04 |
| pfam05977 | DUF894 | 33 | 40 | 3.17 | 7.56E-04 |
| pfam09664 | DUF2399 | 3 | 13 | 3.15 | 8.06E-04 |
| pfam01797 | Transposase_17 | 91 | 84 | 3.13 | 8.73E-04 |
| pfam04539 | Sigma70_r3 | 91 | 84 | 3.13 | 8.73E-04 |
| pfam00317 | Ribonuc_red_lgN | 16 | 25 | 3.12 | 8.95E-04 |
| pfam02272 | DHHA1 | 83 | 78 | 3.12 | 9.13E-04 |
| pfam01471 | PG_binding_1 | 108 | 96 | 3.11 | 9.43E-04 |
| pfam02223 | Thymidylate_kin | 30 | 37 | 3.11 | 9.45E-04 |
| pfam09992 | DUF2233 | 14 | 23 | 3.10 | 9.75E-04 |
| pfam06925 | MGDG_synth | 13 | 22 | 3.09 | 1.01E-03 |
| pfam02465 | FliD_N | 22 | 30 | 3.08 | 1.04E-03 |
| pfam09299 | Mu-transpos_C | 12 | 21 | 3.08 | 1.05E-03 |
| pfam03413 | PepSY | 11 | 20 | 3.07 | 1.08E-03 |
| pfam07559 | FlaE | 2 | 12 | 3.07 | 1.07E-03 |
| pfam10050 | DUF2284 | 2 | 12 | 3.07 | 1.07E-03 |
| pfam00723 | Glyco_hydro_15 | 7 | 16 | 3.05 | 1.16E-03 |
| pfam00850 | Hist_deacetyl | 7 | 16 | 3.05 | 1.16E-03 |
| pfam01436 | NHL | 8 | 17 | 3.05 | 1.15E-03 |
| pfam07811 | TadE | 7 | 16 | 3.05 | 1.16E-03 |
| pfam01583 | APS_kinase | 6 | 15 | 3.04 | 1.17E-03 |
| pfam02288 | Dehydratase_MU | 6 | 15 | 3.04 | 1.17E-03 |
| pfam04434 | SWIM | 5 | 14 | 3.04 | 1.19E-03 |

**Table S3.** continued

| Pfam ID | Name | 40 °C gene count | 55 °C gene count | Z-LOR | *p* value |
| --- | --- | --- | --- | --- | --- |
| pfam03629 | DUF303 | 42 | 7 | -3.03 | 1.21E-03 |
| pfam00570 | HRDC | 32 | 2 | -3.04 | 1.16E-03 |
| pfam00152 | tRNA-synt_2 | 327 | 138 | -3.05 | 1.15E-03 |
| pfam03486 | HI0933_like | 163 | 59 | -3.05 | 1.16E-03 |
| pfam01641 | SelR | 33 | 3 | -3.06 | 1.11E-03 |
| pfam00749 | tRNA-synt_1c | 173 | 63 | -3.10 | 9.52E-04 |
| pfam03062 | MBOAT | 56 | 12 | -3.10 | 9.58E-04 |
| pfam02696 | UPF0061 | 41 | 6 | -3.13 | 8.72E-04 |
| pfam06824 | DUF1237 | 34 | 2 | -3.13 | 8.64E-04 |
| pfam00728 | Glyco_hydro_20 | 44 | 7 | -3.16 | 7.95E-04 |
| pfam02353 | CMAS | 35 | 3 | -3.16 | 7.78E-04 |
| pfam02567 | PhzC-PhzF | 44 | 7 | -3.16 | 7.95E-04 |
| pfam03976 | PPK2 | 39 | 5 | -3.16 | 7.91E-04 |
| pfam01326 | PPDK_N | 144 | 49 | -3.17 | 7.56E-04 |
| pfam03275 | GLF | 52 | 10 | -3.17 | 7.57E-04 |
| pfam02074 | Peptidase_M32 | 55 | 11 | -3.20 | 6.93E-04 |
| pfam01263 | Aldose_epim | 97 | 28 | -3.21 | 6.58E-04 |
| pfam00224 | PK | 118 | 37 | -3.22 | 6.44E-04 |
| pfam01717 | Meth_synt_2 | 37 | 3 | -3.26 | 5.51E-04 |
| pfam00300 | PGAM | 140 | 46 | -3.29 | 4.95E-04 |
| pfam00440 | TetR_N | 273 | 108 | -3.29 | 4.99E-04 |
| pfam02653 | BPD_transp_2 | 720 | 333 | -3.29 | 4.99E-04 |
| pfam04295 | GD_AH_C | 89 | 24 | -3.29 | 4.97E-04 |
| pfam00082 | Peptidase_S8 | 206 | 76 | -3.31 | 4.71E-04 |
| pfam03632 | Glyco_hydro_65m | 87 | 23 | -3.31 | 4.59E-04 |
| pfam03950 | tRNA-synt_1c_C | 44 | 6 | -3.31 | 4.72E-04 |
| pfam05960 | DUF885 | 39 | 2 | -3.33 | 4.28E-04 |
| pfam10022 | DUF2264 | 40 | 4 | -3.34 | 4.25E-04 |
| pfam02896 | PEP-utilizers_C | 179 | 63 | -3.35 | 4.02E-04 |
| pfam00881 | Nitroreductase | 271 | 106 | -3.36 | 3.83E-04 |
| pfam02492 | cobW | 84 | 21 | -3.41 | 3.20E-04 |
| pfam03977 | OAD_beta | 136 | 43 | -3.42 | 3.14E-04 |
| pfam09508 | Lact_bio_phlase | 42 | 2 | -3.44 | 2.89E-04 |
| pfam10566 | Glyco_hydro_97 | 56 | 1 | -3.44 | 2.89E-04 |
| pfam00232 | Glyco_hydro_1 | 298 | 117 | -3.50 | 2.33E-04 |
| pfam00871 | Acetate_kinase | 174 | 59 | -3.51 | 2.27E-04 |
| pfam01053 | Cys_Met_Meta_PP | 326 | 130 | -3.53 | 2.07E-04 |
| pfam06100 | Strep_67kDa_ant | 53 | 8 | -3.53 | 2.11E-04 |
| pfam06874 | FBPase_2 | 61 | 1 | -3.53 | 2.09E-04 |
| pfam07581 | Glug | 45 | 2 | -3.54 | 1.98E-04 |
| pfam04223 | CitF | 56 | 9 | -3.55 | 1.93E-04 |
| pfam02449 | Glyco_hydro_42 | 99 | 26 | -3.56 | 1.88E-04 |
| pfam03313 | SDH_alpha | 87 | 21 | -3.57 | 1.78E-04 |
| pfam03553 | Na_H_antiporter | 67 | 13 | -3.58 | 1.69E-04 |
| pfam01473 | CW_binding_1 | 45 | 3 | -3.61 | 1.51E-04 |
| pfam01228 | Gly_radical | 78 | 17 | -3.62 | 1.45E-04 |

**Table S3.** continued

| Pfam ID | Name | 40 °C gene count | 55 °C gene count | Z-LOR | *p* value |
| --- | --- | --- | --- | --- | --- |
| pfam01055 | Glyco_hydro_31 | 221 | 79 | -3.63 | 1.43E-04 |
| pfam00668 | Condensation | 47 | 4 | -3.67 | 1.22E-04 |
| pfam04204 | HTS | 74 | 15 | -3.68 | 1.16E-04 |
| pfam02836 | Glyco_hydro_2_C | 177 | 58 | -3.72 | 1.01E-04 |
| pfam00255 | GSHPx | 50 | 5 | -3.73 | 9.59E-05 |
| pfam03006 | HlyIII | 50 | 5 | -3.73 | 9.59E-05 |
| pfam08497 | Radical_SAM_N | 49 | 4 | -3.75 | 8.69E-05 |
| pfam05592 | Bac_rhamnosid | 73 | 14 | -3.76 | 8.37E-05 |
| pfam03814 | KdpA | 51 | 5 | -3.78 | 8.00E-05 |
| pfam00248 | Aldo_ket_red | 432 | 177 | -3.80 | 7.21E-05 |
| pfam02518 | HATPase_c | 1169 | 551 | -3.86 | 5.70E-05 |
| pfam07907 | YibE_F | 52 | 4 | -3.88 | 5.29E-05 |
| pfam00145 | DNA_methylase | 156 | 47 | -3.89 | 5.11E-05 |
| pfam10991 | DUF2815 | 54 | 5 | -3.91 | 4.68E-05 |
| pfam06182 | DUF990 | 74 | 13 | -3.94 | 4.02E-05 |
| pfam05738 | Cna_B | 55 | 5 | -3.95 | 3.92E-05 |
| pfam00160 | Pro_isomerase | 72 | 12 | -3.97 | 3.56E-05 |
| pfam03600 | CitMHS | 90 | 19 | -3.97 | 3.61E-05 |
| pfam00486 | Trans_reg_C | 480 | 197 | -3.99 | 3.31E-05 |
| pfam00521 | DNA_topoisoIV | 246 | 85 | -4.05 | 2.56E-05 |
| pfam02503 | PP_kinase | 77 | 13 | -4.09 | 2.18E-05 |
| pfam01144 | CoA_trans | 85 | 16 | -4.10 | 2.08E-05 |
| pfam01638 | HxlR | 85 | 16 | -4.10 | 2.08E-05 |
| pfam02374 | ArsA_ATPase | 102 | 22 | -4.17 | 1.51E-05 |
| pfam10926 | DUF2800 | 84 | 15 | -4.17 | 1.51E-05 |
| pfam06134 | RhaA | 69 | 9 | -4.19 | 1.42E-05 |
| pfam02738 | Ald_Xan_dh_C2 | 153 | 42 | -4.25 | 1.09E-05 |
| pfam00916 | Sulfate_transp | 63 | 5 | -4.26 | 1.01E-05 |
| pfam01235 | Na_Ala_symp | 134 | 34 | -4.26 | 1.02E-05 |
| pfam00933 | Glyco_hydro_3 | 277 | 96 | -4.28 | 9.46E-06 |
| pfam06445 | AraC_E_bind | 107 | 23 | -4.28 | 9.27E-06 |
| pfam02645 | DegV | 204 | 63 | -4.31 | 8.01E-06 |
| pfam01131 | Topoisom_bac | 263 | 89 | -4.33 | 7.62E-06 |
| pfam00122 | E1-E2_ATPase | 439 | 170 | -4.38 | 5.92E-06 |
| pfam00128 | Alpha-amylase | 313 | 111 | -4.38 | 6.00E-06 |
| pfam09479 | Flg_new | 84 | 2 | -4.45 | 4.27E-06 |
| pfam06580 | His_kinase | 326 | 115 | -4.51 | 3.27E-06 |
| pfam07971 | Glyco_hydro_92 | 89 | 2 | -4.53 | 2.88E-06 |
| pfam02321 | OEP | 110 | 21 | -4.63 | 1.82E-06 |
| pfam00083 | Sugar_tr | 87 | 11 | -4.73 | 1.10E-06 |
| pfam00144 | Beta-lactamase | 138 | 31 | -4.73 | 1.12E-06 |
| pfam00990 | GGDEF | 473 | 179 | -4.77 | 9.11E-07 |
| pfam02446 | Glyco_hydro_77 | 84 | 9 | -4.79 | 8.29E-07 |
| pfam02614 | UxaC | 112 | 20 | -4.82 | 7.25E-07 |
| pfam01915 | Glyco_hydro_3_C | 216 | 61 | -4.91 | 4.65E-07 |
| pfam08238 | Sel1 | 96 | 3 | -4.97 | 3.38E-07 |
| pfam00512 | HisKA | 562 | 217 | -4.99 | 3.03E-07 |

**Table S3.** continued

| Pfam ID | Name | 40 °C gene count | 55 °C gene count | Z-LOR | *p* value |
| --- | --- | --- | --- | --- | --- |
| pfam00012 | HSP70 | 209 | 56 | -5.08 | 1.92E-07 |
| pfam00497 | SBP_bac_3 | 299 | 94 | -5.11 | 1.62E-07 |
| pfam07494 | Reg_prop | 93 | 7 | -5.19 | 1.06E-07 |
| pfam00890 | FAD_binding_2 | 288 | 87 | -5.26 | 7.02E-08 |
| pfam05593 | RHS_repeat | 104 | 9 | -5.45 | 2.50E-08 |
| pfam00873 | ACR_tran | 374 | 111 | -6.12 | 4.57E-10 |
| pfam03193 | DUF258 | 236 | 53 | -6.19 | 3.04E-10 |
| pfam04616 | Glyco_hydro_43 | 294 | 76 | -6.22 | 2.54E-10 |
| pfam02368 | Big_2 | 216 | 44 | -6.28 | 1.73E-10 |
| pfam07470 | Glyco_hydro_88 | 156 | 18 | -6.45 | 5.45E-11 |
| pfam00563 | EAL | 315 | 79 | -6.60 | 2.07E-11 |
| pfam07980 | SusD | 198 | 6 | -7.10 | 6.03E-13 |
| pfam00072 | Response_reg | 1387 | 559 | -7.12 | 5.37E-13 |
| pfam01554 | MatE | 440 | 121 | -7.19 | 3.17E-13 |
| pfam02901 | PFL | 266 | 48 | -7.39 | 7.11E-14 |
| pfam00343 | Phosphorylase | 213 | 27 | -7.41 | 6.55E-14 |
| pfam00126 | HTH_1 | 344 | 74 | -7.68 | 7.99E-15 |
| pfam00165 | HTH_AraC | 758 | 242 | -7.98 | 6.66E-16 |
| pfam03466 | LysR_substrate | 453 | 112 | -8.01 | 5.55E-16 |
| pfam07715 | Plug | 328 | 10 | -9.15 | 0.00E+00 |
| pfam00593 | TonB_dep_Rec | 398 | 15 | -10.37 | 0.00E+00 |

^1^ Positive Z-LOR scores indicate Pfams enriched in the thermophilic metagenome and negative Z-LOR scores indicate Pfams enriched in the mesophilic metagenome. Following false discovery rate correction *p*-values ≤ 1.21e-3 were considered to represent statistically significant differences.

^2^Note: Although raw gene counts are provided here, all statistics were generated using gene category proportions (i.e., relative abundances).
