# Supplementary material for: Mesophilic and Thermophilic Conditions Select for Unique but Highly Parallel Microbial Communities to Perform Carboxylate Platform Biomass Conversion
Source: PLoS One. 2012 Jun 22;7(6):e39689. doi: 10.1371/journal.pone.0039689 (PMC3382152; doi:10.1371/journal.pone.0039689)
Supplement: Table S4 — COGs significantly enriched or depleted between the thermophilic and mesophilic metagenomes, as determined by z -normalized log odds ratios (Z-LOR). (DOC) [file pone.0039689.s006.doc]

**Table S4.** COGs significantly enriched or depleted between the thermophilic and mesophilic metagenomes, as determined by *z*-normalized log odds ratios (Z-LOR).^1^

| COG ID | Name | 40 °C gene count ^2^ | 55 °C gene count | Z-LOR | *p* value |
| --- | --- | --- | --- | --- | --- |
| COG1653 | ABC-type sugar transport system, periplasmic component | 337 | 381 | 10.47 | 0.00E+00 |
| COG2826 | Transposase and inactivated derivatives, IS30 family | 62 | 131 | 9.13 | 0.00E+00 |
| COG0675 | Transposase and inactivated derivatives | 21 | 82 | 8.27 | 1.11E-16 |
| COG3328 | Transposase and inactivated derivatives | 113 | 152 | 7.7 | 6.88E-15 |
| COG1203 | Predicted helicases | 35 | 73 | 6.78 | 5.86E-12 |
| COG0438 | Glycosyltransferase | 237 | 226 | 6.59 | 2.26E-11 |
| COG1345 | Flagellar capping protein | 19 | 55 | 6.47 | 4.81E-11 |
| COG1205 | Distinct helicase family with a unique C-terminal domain including a metal-binding cysteine cluster | 36 | 67 | 6.2 | 2.87E-10 |
| COG3933 | Transcriptional antiterminator | 27 | 59 | 6.2 | 2.77E-10 |
| COG0863 | DNA modification methylase | 54 | 80 | 5.98 | 1.14E-09 |
| COG1884 | Methylmalonyl-CoA mutase, N-terminal domain/subunit | 36 | 64 | 5.93 | 1.54E-09 |
| COG0644 | Dehydrogenases (flavoproteins) | 26 | 54 | 5.82 | 2.86E-09 |
| COG2414 | Aldehyde:ferredoxin oxidoreductase | 46 | 71 | 5.78 | 3.79E-09 |
| COG1484 | DNA replication protein | 127 | 134 | 5.76 | 4.21E-09 |
| COG1148 | Heterodisulfide reductase, subunit A and related polyferredoxins | 11 | 40 | 5.73 | 5.05E-09 |
| COG0860 | N-acetylmuramoyl-L-alanine amidase | 160 | 157 | 5.7 | 5.85E-09 |
| COG1940 | Transcriptional regulator/sugar kinase | 171 | 164 | 5.65 | 7.86E-09 |
| COG1191 | DNA-directed RNA polymerase specialized sigma subunit | 185 | 173 | 5.6 | 1.05E-08 |
| COG2002 | Regulators of stationary/sporulation gene expression | 13 | 37 | 5.29 | 6.13E-08 |
| COG2006 | Uncharacterized conserved protein | 18 | 41 | 5.24 | 7.87E-08 |
| COG1796 | DNA polymerase IV (family X) | 7 | 32 | 5.22 | 8.81E-08 |
| COG1082 | Sugar phosphate isomerases/epimerases | 87 | 97 | 5.2 | 9.78E-08 |
| COG1071 | Pyruvate/2-oxoglutarate dehydrogenase complex, dehydrogenase (E1) component, eukaryotic type, alpha subunit | 42 | 61 | 5.15 | 1.30E-07 |
| COG4956 | Integral membrane protein (PIN domain superfamily) | 19 | 41 | 5.15 | 1.32E-07 |
| COG0318 | Acyl-CoA synthetases (AMP-forming)/AMP-acid ligases II | 178 | 161 | 5.14 | 1.36E-07 |
| COG3949 | Uncharacterized membrane protein | 18 | 40 | 5.14 | 1.40E-07 |
| COG2327 | Uncharacterized conserved protein | 9 | 32 | 5.11 | 1.61E-07 |
| COG4636 | Uncharacterized protein conserved in cyanobacteria | 4 | 31 | 5.1 | 1.74E-07 |

**Table S4.** continued

| COG ID | Name | 40 °C gene count | 55 °C gene count | Z-LOR | *p* value |
| --- | --- | --- | --- | --- | --- |
| COG1067 | Predicted ATP-dependent protease | 48 | 65 | 5.06 | 2.11E-07 |
| COG1929 | Glycerate kinase | 52 | 68 | 5.04 | 2.38E-07 |
| COG2152 | Predicted glycosylase | 57 | 71 | 4.94 | 3.84E-07 |
| COG1447 | Phosphotransferase system cellobiose-specific component IIA | 26 | 45 | 4.9 | 4.70E-07 |
| COG1743 | Adenine-specific DNA methylase containing a Zn-ribbon | 13 | 33 | 4.86 | 5.91E-07 |
| COG3589 | Uncharacterized conserved protein | 23 | 42 | 4.86 | 5.77E-07 |
| COG1268 | Uncharacterized conserved protein | 15 | 34 | 4.77 | 9.33E-07 |
| COG0455 | ATPases involved in chromosome partitioning | 22 | 40 | 4.74 | 1.09E-06 |
| COG2189 | Adenine specific DNA methylase Mod | 21 | 39 | 4.72 | 1.16E-06 |
| COG1518 | Uncharacterized protein predicted to be involved in DNA repair | 40 | 55 | 4.71 | 1.26E-06 |
| COG1397 | ADP-ribosylglycohydrolase | 38 | 53 | 4.67 | 1.52E-06 |
| COG1221 | Transcriptional regulators containing an AAA-type ATPase domain and a DNA-binding domain | 21 | 38 | 4.61 | 2.05E-06 |
| COG1351 | Predicted alternative thymidylate synthase | 12 | 30 | 4.61 | 1.98E-06 |
| COG1387 | Histidinol phosphatase and related hydrolases of the PHP family | 59 | 69 | 4.6 | 2.09E-06 |
| COG1598 | Uncharacterized conserved protein | 5 | 24 | 4.53 | 2.92E-06 |
| COG1961 | Site-specific recombinases, DNA invertase Pin homologs | 131 | 120 | 4.53 | 3.01E-06 |
| COG1440 | Phosphotransferase system cellobiose-specific component IIB | 23 | 39 | 4.52 | 3.14E-06 |
| COG1762 | Phosphotransferase system mannitol/fructose-specific IIA domain (Ntr-type) | 51 | 62 | 4.52 | 3.07E-06 |
| COG3547 | Transposase and inactivated derivatives | 29 | 44 | 4.5 | 3.40E-06 |
| COG1388 | FOG: LysM repeat | 12 | 29 | 4.49 | 3.53E-06 |
| COG1480 | Predicted membrane-associated HD superfamily hydrolase | 34 | 48 | 4.48 | 3.73E-06 |
| COG1922 | Teichoic acid biosynthesis proteins | 20 | 36 | 4.47 | 3.88E-06 |
| COG2421 | Predicted acetamidase/formamidase | 10 | 27 | 4.46 | 4.02E-06 |
| COG2445 | Uncharacterized conserved protein | 2 | 27 | 4.45 | 4.26E-06 |
| COG1468 | RecB family exonuclease | 5 | 23 | 4.43 | 4.73E-06 |
| COG2244 | Membrane protein involved in the export of O-antigen and teichoic acid | 17 | 33 | 4.43 | 4.70E-06 |
| COG5012 | Predicted cobalamin binding protein | 25 | 40 | 4.43 | 4.72E-06 |
| COG0641 | Arylsulfatase regulator (Fe-S oxidoreductase) | 62 | 69 | 4.38 | 5.94E-06 |

**Table S4.** continued

| COG ID | Name | 40 °C gene count | 55 °C gene count | Z-LOR | *p* value |
| --- | --- | --- | --- | --- | --- |
| COG3875 | Uncharacterized conserved protein | 34 | 47 | 4.37 | 6.30E-06 |
| COG1595 | DNA-directed RNA polymerase specialized sigma subunit, sigma24 homolog | 250 | 196 | 4.36 | 6.40E-06 |
| COG3409 | Putative peptidoglycan-binding domain-containing protein | 28 | 42 | 4.36 | 6.37E-06 |
| COG3856 | Uncharacterized conserved protein (small basic protein) | 4 | 22 | 4.35 | 6.83E-06 |
| COG0407 | Uroporphyrinogen-III decarboxylase | 26 | 40 | 4.33 | 7.54E-06 |
| COG4962 | Flp pilus assembly protein, ATPase CpaF | 60 | 67 | 4.33 | 7.42E-06 |
| COG2172 | Anti-sigma regulatory factor (Ser/Thr protein kinase) | 36 | 48 | 4.3 | 8.71E-06 |
| COG1873 | Uncharacterized conserved protein | 6 | 22 | 4.25 | 1.06E-05 |
| COG3323 | Uncharacterized protein conserved in bacteria | 4 | 21 | 4.25 | 1.08E-05 |
| COG4666 | TRAP-type uncharacterized transport system, fused permease components | 27 | 40 | 4.23 | 1.19E-05 |
| COG1669 | Predicted nucleotidyltransferases | 3 | 21 | 4.22 | 1.22E-05 |
| COG2045 | Phosphosulfolactate phosphohydrolase and related enzymes | 11 | 26 | 4.22 | 1.20E-05 |
| COG0689 | RNase PH | 19 | 33 | 4.21 | 1.30E-05 |
| COG1583 | Uncharacterized protein predicted to be involved in DNA repair (RAMP superfamily) | 2 | 23 | 4.21 | 1.29E-05 |
| COG0728 | Uncharacterized membrane protein, putative virulence factor | 31 | 43 | 4.19 | 1.41E-05 |
| COG0022 | Pyruvate/2-oxoglutarate dehydrogenase complex, dehydrogenase (E1) component, eukaryotic type, beta subunit | 58 | 64 | 4.18 | 1.46E-05 |
| COG3894 | Uncharacterized metal-binding protein | 30 | 42 | 4.17 | 1.55E-05 |
| COG4584 | Transposase and inactivated derivatives | 45 | 54 | 4.17 | 1.52E-05 |
| COG5280 | Phage-related minor tail protein | 7 | 22 | 4.16 | 1.60E-05 |
| COG3414 | Phosphotransferase system, galactitol-specific IIB component | 4 | 20 | 4.14 | 1.72E-05 |
| COG3773 | Cell wall hydrolyses involved in spore germination | 60 | 65 | 4.13 | 1.81E-05 |
| COG3879 | Uncharacterized protein conserved in bacteria | 22 | 35 | 4.13 | 1.81E-05 |
| COG0010 | Arginase/agmatinase/formimionoglutamate hydrolase, arginase family | 51 | 58 | 4.11 | 2.02E-05 |
| COG4856 | Uncharacterized protein conserved in bacteria | 12 | 26 | 4.1 | 2.02E-05 |
| COG0472 | UDP-N-acetylmuramyl pentapeptide phosphotransferase/UDP-N-acetylglucosamine-1-phosphate transferase | 77 | 77 | 4.09 | 2.14E-05 |

**Table S4.** continued

| COG ID | Name | 40 °C gene count | 55 °C gene count | Z-LOR | *p* value |
| --- | --- | --- | --- | --- | --- |
| COG0835 | Chemotaxis signal transduction protein | 73 | 74 | 4.08 | 2.25E-05 |
| COG1220 | ATP-dependent protease HslVU (ClpYQ), ATPase subunit | 26 | 38 | 4.08 | 2.23E-05 |
| COG5401 | Spore germination protein | 8 | 22 | 4.05 | 2.59E-05 |
| COG1776 | Chemotaxis protein CheC, inhibitor of MCP methylation | 30 | 41 | 4.04 | 2.62E-05 |
| COG1978 | Uncharacterized protein conserved in bacteria | 3 | 19 | 4.03 | 2.76E-05 |
| COG5421 | Transposase | 14 | 27 | 4 | 3.22E-05 |
| COG3711 | Transcriptional antiterminator | 73 | 73 | 3.98 | 3.39E-05 |
| COG1960 | Acyl-CoA dehydrogenases | 148 | 124 | 3.96 | 3.69E-05 |
| COG1401 | GTPase subunit of restriction endonuclease | 3 | 18 | 3.93 | 4.24E-05 |
| COG0843 | Heme/copper-type cytochrome/quinol oxidases, subunit 1 | 35 | 44 | 3.92 | 4.40E-05 |
| COG3314 | Uncharacterized protein conserved in bacteria | 4 | 18 | 3.91 | 4.54E-05 |
| COG4365 | Uncharacterized protein conserved in bacteria | 4 | 18 | 3.91 | 4.54E-05 |
| COG4508 | Uncharacterized protein conserved in bacteria | 4 | 18 | 3.91 | 4.54E-05 |
| COG0683 | ABC-type branched-chain amino acid transport systems, periplasmic component | 118 | 103 | 3.88 | 5.17E-05 |
| COG0732 | Restriction endonuclease S subunits | 28 | 38 | 3.87 | 5.36E-05 |
| COG2120 | Uncharacterized proteins, LmbE homologs | 15 | 27 | 3.87 | 5.38E-05 |
| COG1315 | Predicted polymerase, most proteins contain PALM domain, HD hydrolase domain and Zn-ribbon domain | 45 | 51 | 3.84 | 6.25E-05 |
| COG3044 | Predicted ATPase of the ABC class | 3 | 17 | 3.82 | 6.60E-05 |
| COG1514 | 2'-5' RNA ligase | 19 | 30 | 3.81 | 7.04E-05 |
| COG3458 | Acetyl esterase (deacetylase) | 19 | 30 | 3.81 | 7.04E-05 |
| COG1457 | Purine-cytosine permease and related proteins | 10 | 22 | 3.8 | 7.35E-05 |
| COG2185 | Methylmalonyl-CoA mutase, C-terminal domain/subunit (cobalamin-binding) | 10 | 22 | 3.8 | 7.35E-05 |
| COG4722 | Phage-related protein | 10 | 22 | 3.8 | 7.35E-05 |
| COG0395 | ABC-type sugar transport system, permease component | 794 | 509 | 3.79 | 7.53E-05 |
| COG1210 | UDP-glucose pyrophosphorylase | 59 | 61 | 3.79 | 7.42E-05 |
| COG1633 | Uncharacterized conserved protein | 4 | 17 | 3.79 | 7.53E-05 |
| COG0535 | Predicted Fe-S oxidoreductases | 69 | 68 | 3.77 | 8.05E-05 |
| COG2119 | Predicted membrane protein | 8 | 20 | 3.77 | 8.28E-05 |

**Table S4.** continued

| COG ID | Name | 40 °C gene count | 55 °C gene count | Z-LOR | *p* value |
| --- | --- | --- | --- | --- | --- |
| COG0619 | ABC-type cobalt transport system, permease component CbiQ and related transporters | 57 | 59 | 3.74 | 9.34E-05 |
| COG1700 | Uncharacterized conserved protein | 2 | 17 | 3.74 | 9.03E-05 |
| COG2231 | Uncharacterized protein related to Endonuclease III | 2 | 17 | 3.74 | 9.03E-05 |
| COG0182 | Predicted translation initiation factor 2B subunit, eIF-2B alpha/beta/delta family | 22 | 32 | 3.73 | 9.44E-05 |
| COG2225 | Malate synthase | 15 | 26 | 3.73 | 9.58E-05 |
| COG1293 | Predicted RNA-binding protein homologous to eukaryotic snRNP | 56 | 58 | 3.71 | 1.05E-04 |
| COG4096 | Type I site-specific restriction-modification system, R (restriction) subunit and related helicases | 40 | 46 | 3.7 | 1.09E-04 |
| COG0247 | Fe-S oxidoreductase | 48 | 52 | 3.69 | 1.10E-04 |
| COG3723 | Recombinational DNA repair protein (RecE pathway) | 13 | 24 | 3.69 | 1.10E-04 |
| COG2208 | Serine phosphatase RsbU, regulator of sigma subunit | 30 | 38 | 3.67 | 1.22E-04 |
| COG2021 | Homoserine acetyltransferase | 11 | 22 | 3.66 | 1.25E-04 |
| COG3535 | Uncharacterized conserved protein | 11 | 22 | 3.66 | 1.25E-04 |
| COG1914 | Mn2+ and Fe2+ transporters of the NRAMP family | 46 | 50 | 3.64 | 1.39E-04 |
| COG2179 | Predicted hydrolase of the HAD superfamily | 18 | 28 | 3.64 | 1.34E-04 |
| COG0119 | Isopropylmalate/homocitrate/citramalate synthases | 169 | 133 | 3.62 | 1.46E-04 |
| COG0123 | Deacetylases, including yeast histone deacetylase and acetoin utilization protein | 8 | 19 | 3.62 | 1.49E-04 |
| COG1404 | Subtilisin-like serine proteases | 28 | 36 | 3.61 | 1.51E-04 |
| COG1943 | Transposase and inactivated derivatives | 63 | 62 | 3.6 | 1.61E-04 |
| COG1144 | Pyruvate:ferredoxin oxidoreductase and related 2-oxoacid:ferredoxin oxidoreductases, delta subunit | 3 | 15 | 3.59 | 1.67E-04 |
| COG1455 | Phosphotransferase system cellobiose-specific component IIC | 122 | 102 | 3.58 | 1.71E-04 |
| COG4848 | Uncharacterized protein conserved in bacteria | 6 | 17 | 3.58 | 1.71E-04 |
| COG4965 | Flp pilus assembly protein TadB | 15 | 25 | 3.58 | 1.70E-04 |
| COG0686 | Alanine dehydrogenase | 21 | 30 | 3.57 | 1.78E-04 |
| COG5598 | Trimethylamine:corrinoid methyltransferase | 1 | 20 | 3.57 | 1.80E-04 |
| COG1861 | Spore coat polysaccharide biosynthesis protein F, CMP-KDO synthetase homolog | 5 | 16 | 3.56 | 1.87E-04 |

**Table S4.** continued

| COG ID | Name | 40 °C gene count | 55 °C gene count | Z-LOR | *p* value |
| --- | --- | --- | --- | --- | --- |
| COG0726 | Predicted xylanase/chitin deacetylase | 121 | 101 | 3.55 | 1.91E-04 |
| COG3881 | Uncharacterized protein conserved in bacteria | 2 | 15 | 3.55 | 1.91E-04 |
| COG1353 | Predicted hydrolase of the HD superfamily (permuted catalytic motifs) | 1 | 19 | 3.51 | 2.22E-04 |
| COG2876 | 3-deoxy-D-arabino-heptulosonate 7-phosphate (DAHP) synthase | 70 | 66 | 3.5 | 2.32E-04 |
| COG1149 | MinD superfamily P-loop ATPase containing an inserted ferredoxin domain | 52 | 53 | 3.48 | 2.55E-04 |
| COG4604 | ABC-type enterochelin transport system, ATPase component | 9 | 19 | 3.48 | 2.55E-04 |
| COG4392 | Predicted membrane protein | 3 | 14 | 3.46 | 2.72E-04 |
| COG4825 | Uncharacterized membrane-anchored protein conserved in bacteria | 3 | 14 | 3.46 | 2.72E-04 |
| COG3666 | Transposase and inactivated derivatives | 27 | 34 | 3.45 | 2.78E-04 |
| COG4939 | Major membrane immunogen, membrane-anchored lipoprotein | 2 | 14 | 3.45 | 2.84E-04 |
| COG0739 | Membrane proteins related to metalloendopeptidases | 123 | 101 | 3.44 | 2.87E-04 |
| COG1334 | Uncharacterized flagellar protein FlaG | 7 | 17 | 3.44 | 2.87E-04 |
| COG4821 | Uncharacterized protein containing SIS (Sugar ISomerase) phosphosugar binding domain | 7 | 17 | 3.44 | 2.87E-04 |
| COG0125 | Thymidylate kinase | 31 | 37 | 3.43 | 2.97E-04 |
| COG2390 | Transcriptional regulator, contains sigma factor-related N-terminal domain | 35 | 40 | 3.43 | 3.07E-04 |
| COG3664 | Beta-xylosidase | 21 | 29 | 3.43 | 3.05E-04 |
| COG4606 | ABC-type enterochelin transport system, permease component | 15 | 24 | 3.43 | 3.01E-04 |
| COG2385 | Sporulation protein and related proteins | 104 | 88 | 3.4 | 3.38E-04 |
| COG0391 | Uncharacterized conserved protein | 34 | 39 | 3.39 | 3.43E-04 |
| COG0432 | Uncharacterized conserved protein | 13 | 22 | 3.39 | 3.51E-04 |
| COG4496 | Uncharacterized protein conserved in bacteria | 13 | 22 | 3.39 | 3.51E-04 |
| COG4972 | Tfp pilus assembly protein, ATPase PilM | 13 | 22 | 3.39 | 3.51E-04 |
| COG1343 | Uncharacterized protein predicted to be involved in DNA repair | 12 | 21 | 3.37 | 3.78E-04 |
| COG3829 | Transcriptional regulator containing PAS, AAA-type ATPase, and DNA-binding domains | 204 | 151 | 3.34 | 4.17E-04 |
| COG0600 | ABC-type nitrate/sulfonate/bicarbonate transport system, permease component | 147 | 115 | 3.33 | 4.42E-04 |

**Table S4.** continued

| COG ID | Name | 40 °C gene count | 55 °C gene count | Z-LOR | *p* value |
| --- | --- | --- | --- | --- | --- |
| COG1030 | Membrane-bound serine protease (ClpP class) | 10 | 19 | 3.33 | 4.33E-04 |
| COG1206 | NAD(FAD)-utilizing enzyme possibly involved in translation | 40 | 43 | 3.33 | 4.33E-04 |
| COG3599 | Cell division initiation protein | 10 | 19 | 3.33 | 4.33E-04 |
| COG5441 | Uncharacterized conserved protein | 2 | 13 | 3.33 | 4.31E-04 |
| COG2718 | Uncharacterized conserved protein | 17 | 25 | 3.32 | 4.43E-04 |
| COG3033 | Tryptophanase | 9 | 18 | 3.31 | 4.62E-04 |
| COG1575 | 1,4-dihydroxy-2-naphthoate octaprenyltransferase | 31 | 36 | 3.3 | 4.81E-04 |
| COG0718 | Uncharacterized protein conserved in bacteria | 21 | 28 | 3.28 | 5.18E-04 |
| COG1994 | Zn-dependent proteases | 7 | 16 | 3.28 | 5.21E-04 |
| COG5405 | ATP-dependent protease HslVU (ClpYQ), peptidase subunit | 15 | 23 | 3.27 | 5.29E-04 |
| COG1078 | HD superfamily phosphohydrolases | 25 | 31 | 3.25 | 5.71E-04 |
| COG1331 | Highly conserved protein containing a thioredoxin domain | 65 | 60 | 3.24 | 6.06E-04 |
| COG1403 | Restriction endonuclease | 5 | 14 | 3.24 | 5.94E-04 |
| COG3144 | Flagellar hook-length control protein | 5 | 14 | 3.24 | 5.94E-04 |
| COG4399 | Uncharacterized protein conserved in bacteria | 5 | 14 | 3.24 | 5.94E-04 |
| COG0849 | Actin-like ATPase involved in cell division | 55 | 53 | 3.23 | 6.12E-04 |
| COG0145 | N-methylhydantoinase A/acetone carboxylase, beta subunit | 44 | 45 | 3.22 | 6.50E-04 |
| COG2719 | Uncharacterized conserved protein | 19 | 26 | 3.22 | 6.33E-04 |
| COG1208 | Nucleoside-diphosphate-sugar pyrophosphorylase involved in lipopolysaccharide biosynthesis/translation initiation factor 2B, gamma/epsilon subunits (eIF-2Bgamma/eIF-2Bepsilon) | 61 | 57 | 3.21 | 6.61E-04 |
| COG2367 | Beta-lactamase class A | 18 | 25 | 3.2 | 6.98E-04 |
| COG3290 | Signal transduction histidine kinase regulating citrate/malate metabolism | 47 | 47 | 3.2 | 6.96E-04 |
| COG5322 | Predicted dehydrogenase | 1 | 14 | 3.19 | 7.20E-04 |
| COG0640 | Predicted transcriptional regulators | 79 | 69 | 3.18 | 7.35E-04 |
| COG0105 | Nucleoside diphosphate kinase | 17 | 24 | 3.17 | 7.68E-04 |
| COG0433 | Predicted ATPase | 17 | 24 | 3.17 | 7.68E-04 |
| COG0618 | Exopolyphosphatase-related proteins | 38 | 40 | 3.14 | 8.53E-04 |

**Table S4.** continued

| COG ID | Name | 40 °C gene count | 55 °C gene count | Z-LOR | *p* value |
| --- | --- | --- | --- | --- | --- |
| COG1379 | Uncharacterized conserved protein | 16 | 23 | 3.14 | 8.45E-04 |
| COG1728 | Uncharacterized protein conserved in bacteria | 9 | 17 | 3.14 | 8.39E-04 |
| COG1098 | Predicted RNA binding protein (contains ribosomal protein S1 domain) | 15 | 22 | 3.11 | 9.27E-04 |
| COG1734 | DnaK suppressor protein | 15 | 22 | 3.11 | 9.27E-04 |
| COG1815 | Flagellar basal body protein | 15 | 22 | 3.11 | 9.27E-04 |
| COG2137 | Uncharacterized protein conserved in bacteria | 15 | 22 | 3.11 | 9.27E-04 |
| COG3267 | Type II secretory pathway, component ExeA (predicted ATPase) | 15 | 22 | 3.11 | 9.27E-04 |
| COG1358 | Ribosomal protein HS6-type (S12/L30/L7a) | 14 | 21 | 3.09 | 1.01E-03 |
| COG4851 | Protein involved in sex pheromone biosynthesis | 6 | 14 | 3.09 | 1.01E-03 |
| COG4359 | Uncharacterized conserved protein, possibly involved in methylthioadenosine recycling | 2 | 11 | 3.08 | 1.05E-03 |
| COG4952 | Predicted sugar isomerase | 2 | 11 | 3.08 | 1.05E-03 |
| COG4968 | Tfp pilus assembly protein PilE | 5 | 13 | 3.07 | 1.08E-03 |
| COG0075 | Serine-pyruvate aminotransferase/archaeal aspartate aminotransferase | 43 | 43 | 3.06 | 1.12E-03 |
| COG1014 | Pyruvate:ferredoxin oxidoreductase and related 2-oxoacid:ferredoxin oxidoreductases, gamma subunit | 99 | 81 | 3.06 | 1.10E-03 |
| COG1196 | Chromosome segregation ATPases | 154 | 116 | 3.06 | 1.11E-03 |
| COG0864 | Predicted transcriptional regulators containing the CopG/Arc/MetJ DNA-binding domain and a metal-binding domain | 4 | 12 | 3.04 | 1.16E-03 |
| COG1612 | Uncharacterized protein required for cytochrome oxidase assembly | 4 | 12 | 3.04 | 1.16E-03 |
| COG1703 | Putative periplasmic protein kinase ArgK and related GTPases of G3E family | 18 | 24 | 3.04 | 1.19E-03 |
| COG3587 | Restriction endonuclease | 56 | 52 | 3.04 | 1.19E-03 |
| COG1749 | Flagellar hook protein FlgE | 35 | 37 | 3.03 | 1.22E-03 |
| COG1641 | Uncharacterized conserved protein | 42 | 42 | 3.02 | 1.26E-03 |
| COG4756 | Predicted cation transporter | 1 | 12 | 3.02 | 1.26E-03 |
| COG1024 | Enoyl-CoA hydratase/carnithine racemase | 74 | 64 | 3.01 | 1.30E-03 |
| COG1954 | Glycerol-3-phosphate responsive antiterminator (mRNA-binding) | 22 | 27 | 3.01 | 1.31E-03 |
| COG5662 | Predicted transmembrane transcriptional regulator (anti-sigma factor) | 3 | 11 | 3.01 | 1.32E-03 |
| COG1317 | Flagellar biosynthesis/type III secretory pathway protein | 10 | 17 | 2.99 | 1.41E-03 |

**Table S4.** continued

| COG ID | Name | 40 °C gene count | 55 °C gene count | Z-LOR | *p* value |
| --- | --- | --- | --- | --- | --- |
| COG4242 | Cyanophycinase and related exopeptidases | 10 | 17 | 2.99 | 1.41E-03 |
| COG3387 | Glucoamylase and related glycosyl hydrolases | 16 | 22 | 2.98 | 1.46E-03 |
| COG0344 | Predicted membrane protein | 29 | 32 | 2.96 | 1.56E-03 |
| COG0661 | Predicted unusual protein kinase | 29 | 32 | 2.96 | 1.56E-03 |
| COG1333 | ResB protein required for cytochrome c biosynthesis | 9 | 16 | 2.96 | 1.52E-03 |
| COG3870 | Uncharacterized protein conserved in bacteria | 15 | 21 | 2.95 | 1.61E-03 |
| COG4463 | Transcriptional repressor of class III stress genes | 15 | 21 | 2.95 | 1.61E-03 |
| COG0529 | Adenylylsulfate kinase and related kinases | 8 | 15 | 2.94 | 1.63E-03 |
| COG0715 | ABC-type nitrate/sulfonate/bicarbonate transport systems, periplasmic components | 115 | 90 | 2.94 | 1.62E-03 |
| COG2046 | ATP sulfurylase (sulfate adenylyltransferase) | 8 | 15 | 2.94 | 1.63E-03 |
| COG4200 | Uncharacterized protein conserved in bacteria | 2 | 10 | 2.93 | 1.70E-03 |
| COG4469 | Competence protein | 2 | 10 | 2.93 | 1.70E-03 |
| COG0690 | Preprotein translocase subunit SecE | 7 | 14 | 2.92 | 1.74E-03 |
| COG0746 | Molybdopterin-guanine dinucleotide biosynthesis protein A | 7 | 14 | 2.92 | 1.74E-03 |
| COG1267 | Phosphatidylglycerophosphatase A and related proteins | 7 | 14 | 2.92 | 1.74E-03 |
| COG1475 | Predicted transcriptional regulators | 89 | 73 | 2.92 | 1.75E-03 |
| COG2052 | Uncharacterized protein conserved in bacteria | 24 | 28 | 2.92 | 1.73E-03 |
| COG2971 | Predicted N-acetylglucosamine kinase | 14 | 20 | 2.92 | 1.78E-03 |
| COG2998 | ABC-type tungstate transport system, permease component | 7 | 14 | 2.92 | 1.74E-03 |
| COG0629 | Single-stranded DNA-binding protein | 83 | 69 | 2.91 | 1.79E-03 |
| COG2856 | Predicted Zn peptidase | 19 | 24 | 2.91 | 1.82E-03 |
| COG1024 | Enoyl-CoA hydratase/carnithine racemase | 74 | 64 | 3.01 | 1.30E-03 |
| COG1963 | Uncharacterized protein conserved in bacteria | 6 | 13 | 2.9 | 1.85E-03 |
| COG0558 | Phosphatidylglycerophosphate synthase | 31 | 33 | 2.89 | 1.95E-03 |
| COG1868 | Flagellar motor switch protein | 55 | 50 | 2.89 | 1.95E-03 |
| COG2145 | Hydroxyethylthiazole kinase, sugar kinase family | 42 | 41 | 2.89 | 1.91E-03 |
| COG5581 | Predicted glycosyltransferase | 5 | 12 | 2.88 | 1.97E-03 |
| COG5614 | Bacteriophage head-tail adaptor | 5 | 12 | 2.88 | 1.97E-03 |
| COG2131 | Deoxycytidylate deaminase | 30 | 32 | 2.85 | 2.20E-03 |
| COG3583 | Uncharacterized protein conserved in bacteria | 30 | 32 | 2.85 | 2.20E-03 |

**Table S4.** continued

| COG ID | Name | 40 °C gene count | 55 °C gene count | Z-LOR | *p* value |
| --- | --- | --- | --- | --- | --- |
| COG4626 | Phage terminase-like protein, large subunit | 128 | 97 | 2.84 | 2.27E-03 |
| COG3442 | Predicted glutamine amidotransferase | 11 | 17 | 2.83 | 2.34E-03 |
| COG0071 | Molecular chaperone (small heat shock protein) | 40 | 39 | 2.82 | 2.42E-03 |
| COG1489 | DNA-binding protein, stimulates sugar fermentation | 29 | 31 | 2.81 | 2.48E-03 |
| COG2730 | Endoglucanase | 25 | 28 | 2.81 | 2.49E-03 |
| COG2182 | Maltose-binding periplasmic proteins/domains | 71 | 60 | 2.8 | 2.55E-03 |
| COG2608 | Copper chaperone | 36 | 36 | 2.8 | 2.58E-03 |
| COG3872 | Predicted metal-dependent enzyme | 43 | 41 | 2.8 | 2.54E-03 |
| COG0622 | Predicted phosphoesterase | 49 | 45 | 2.78 | 2.71E-03 |
| COG2739 | Uncharacterized protein conserved in bacteria | 15 | 20 | 2.77 | 2.78E-03 |
| COG3941 | Mu-like prophage protein | 2 | 9 | 2.77 | 2.83E-03 |
| COG4347 | Predicted membrane protein | 2 | 9 | 2.77 | 2.83E-03 |
| COG4502 | Uncharacterized protein conserved in bacteria | 2 | 9 | 2.77 | 2.83E-03 |
| COG1721 | Uncharacterized conserved protein (some members contain a von Willebrand factor type A (vWA) domain) | 35 | 35 | 2.76 | 2.91E-03 |
| COG0146 | N-methylhydantoinase B/acetone carboxylase, alpha subunit | 8 | 14 | 2.75 | 2.98E-03 |
| COG1141 | Ferredoxin | 14 | 19 | 2.74 | 3.08E-03 |
| COG2766 | Putative Ser protein kinase | 31 | 32 | 2.74 | 3.05E-03 |
| COG0011 | Uncharacterized conserved protein | 7 | 13 | 2.73 | 3.20E-03 |
| COG0508 | Pyruvate/2-oxoglutarate dehydrogenase complex, dihydrolipoamide acyltransferase (E2) component, and related enzymes | 103 | 80 | 2.73 | 3.18E-03 |
| COG1426 | Uncharacterized protein conserved in bacteria | 7 | 13 | 2.73 | 3.20E-03 |
| COG2359 | Uncharacterized protein conserved in bacteria | 23 | 26 | 2.73 | 3.15E-03 |
| COG4466 | Uncharacterized protein conserved in bacteria | 7 | 13 | 2.73 | 3.20E-03 |
| COG5578 | Predicted integral membrane protein | 7 | 13 | 2.73 | 3.20E-03 |
| COG1344 | Flagellin and related hook-associated proteins | 148 | 108 | 2.72 | 3.26E-03 |
| COG2337 | Growth inhibitor | 34 | 34 | 2.72 | 3.28E-03 |
| COG0673 | Predicted dehydrogenases and related proteins | 275 | 184 | 2.71 | 3.41E-03 |
| COG3090 | TRAP-type C4-dicarboxylate transport system, small permease component | 13 | 18 | 2.71 | 3.41E-03 |
| COG4826 | Serine protease inhibitor | 13 | 18 | 2.71 | 3.41E-03 |

**Table S4.** continued

| COG ID | Name | 40 °C gene count | 55 °C gene count | Z-LOR | *p* value |
| --- | --- | --- | --- | --- | --- |
| COG0421 | Spermidine synthase | 47 | 43 | 2.7 | 3.44E-03 |
| COG1811 | Uncharacterized membrane protein, possible Na+ channel or pump | 30 | 31 | 2.7 | 3.44E-03 |
| COG3679 | Uncharacterized conserved protein | 6 | 12 | 2.7 | 3.42E-03 |
| COG3693 | Beta-1,4-xylanase | 50 | 45 | 2.7 | 3.51E-03 |
| COG4843 | Uncharacterized protein conserved in bacteria | 6 | 12 | 2.7 | 3.42E-03 |
| COG1009 | NADH:ubiquinone oxidoreductase subunit 5 (chain L)/Multisubunit Na+/H+ antiporter, MnhA subunit | 65 | 55 | 2.69 | 3.60E-03 |
| COG2316 | Predicted hydrolase (HD superfamily) | 22 | 25 | 2.69 | 3.54E-03 |
| COG1615 | Uncharacterized conserved protein | 33 | 33 | 2.68 | 3.70E-03 |
| COG3740 | Phage head maturation protease | 12 | 17 | 2.67 | 3.77E-03 |
| COG3880 | Uncharacterized protein with conserved CXXC pairs | 17 | 21 | 2.67 | 3.81E-03 |
| COG0365 | Acyl-coenzyme A synthetases/AMP-(fatty) acid ligases | 64 | 54 | 2.65 | 4.04E-03 |
| COG0647 | Predicted sugar phosphatases of the HAD superfamily | 21 | 24 | 2.65 | 3.99E-03 |
| COG1122 | ABC-type cobalt transport system, ATPase component | 184 | 129 | 2.65 | 4.03E-03 |
| COG1559 | Predicted periplasmic solute-binding protein | 52 | 46 | 2.65 | 4.00E-03 |
| COG1041 | Predicted DNA modification methylase | 3 | 9 | 2.64 | 4.18E-03 |
| COG1259 | Uncharacterized conserved protein | 3 | 9 | 2.64 | 4.18E-03 |
| COG1942 | Uncharacterized protein, 4-oxalocrotonate tautomerase homolog | 3 | 9 | 2.64 | 4.18E-03 |
| COG4127 | Uncharacterized conserved protein | 3 | 9 | 2.64 | 4.18E-03 |
| COG4241 | Predicted membrane protein | 3 | 9 | 2.64 | 4.18E-03 |
| COG3601 | Predicted membrane protein | 16 | 20 | 2.63 | 4.26E-03 |
| COG1847 | Predicted RNA-binding protein | 28 | 29 | 2.62 | 4.39E-03 |
| COG4465 | Pleiotropic transcriptional repressor | 28 | 29 | 2.62 | 4.39E-03 |
| COG1735 | Predicted metal-dependent hydrolase with the TIM-barrel fold | 10 | 15 | 2.61 | 4.55E-03 |
| COG5412 | Phage-related protein | 20 | 23 | 2.61 | 4.48E-03 |
| COG2240 | Pyridoxal/pyridoxine/pyridoxamine kinase | 31 | 4 | -2.61 | 4.48E-03 |
| COG2957 | Peptidylarginine deiminase and related enzymes | 31 | 4 | -2.61 | 4.48E-03 |
| COG4213 | ABC-type xylose transport system, periplasmic component | 122 | 39 | -2.62 | 4.43E-03 |
| COG0402 | Cytosine deaminase and related metal-dependent hydrolases | 115 | 36 | -2.63 | 4.26E-03 |
| COG3604 | Transcriptional regulator containing GAF, AAA-type ATPase, and DNA binding domains | 51 | 11 | -2.63 | 4.25E-03 |

**Table S4.** continued

| COG ID | Name | 40 °C gene count | 55 °C gene count | Z-LOR | *p* value |
| --- | --- | --- | --- | --- | --- |
| COG4948 | L-alanine-DL-glutamate epimerase and related enzymes of enolase superfamily | 127 | 41 | -2.63 | 4.33E-03 |
| COG1048 | Aconitase A | 198 | 71 | -2.65 | 4.02E-03 |
| COG2272 | Carboxylesterase type B | 40 | 7 | -2.65 | 4.08E-03 |
| COG0366 | Glycosidases | 212 | 77 | -2.66 | 3.92E-03 |
| COG0545 | FKBP-type peptidyl-prolyl cis-trans isomerases 1 | 29 | 1 | -2.66 | 3.88E-03 |
| COG1643 | HrpA-like helicases | 43 | 8 | -2.66 | 3.95E-03 |
| COG3023 | Negative regulator of beta-lactamase expression | 32 | 4 | -2.68 | 3.70E-03 |
| COG1757 | Na+/H+ antiporter | 52 | 11 | -2.69 | 3.52E-03 |
| COG3405 | Endoglucanase Y | 35 | 5 | -2.69 | 3.56E-03 |
| COG3264 | Small-conductance mechanosensitive channel | 38 | 6 | -2.7 | 3.45E-03 |
| COG0588 | Phosphoglycerate mutase 1 | 30 | 3 | -2.71 | 3.32E-03 |
| COG1448 | Aspartate/tyrosine/aromatic aminotransferase | 28 | 2 | -2.71 | 3.41E-03 |
| COG4932 | Predicted outer membrane protein | 28 | 2 | -2.71 | 3.41E-03 |
| COG1502 | Phosphatidylserine/phosphatidylglycerophosphate/cardiolipin synthases and related enzymes | 97 | 28 | -2.72 | 3.27E-03 |
| COG1883 | Na+-transporting methylmalonyl-CoA/oxaloacetate decarboxylase, beta subunit | 134 | 43 | -2.73 | 3.21E-03 |
| COG4289 | Uncharacterized protein conserved in bacteria | 33 | 4 | -2.74 | 3.06E-03 |
| COG3634 | Alkyl hydroperoxide reductase, large subunit | 72 | 18 | -2.76 | 2.89E-03 |
| COG1373 | Predicted ATPase (AAA+ superfamily) | 39 | 6 | -2.77 | 2.84E-03 |
| COG1528 | Ferritin-like protein | 31 | 3 | -2.77 | 2.78E-03 |
| COG4667 | Predicted esterase of the alpha-beta hydrolase superfamily | 31 | 3 | -2.77 | 2.78E-03 |
| COG0270 | Site-specific DNA methylase | 126 | 39 | -2.8 | 2.53E-03 |
| COG1501 | Alpha-glucosidases, family 31 of glycosyl hydrolases | 241 | 88 | -2.8 | 2.57E-03 |
| COG1156 | Archaeal/vacuolar-type H+-ATPase subunit B | 131 | 41 | -2.81 | 2.49E-03 |
| COG2199 | FOG: GGDEF domain | 318 | 122 | -2.81 | 2.49E-03 |
| COG4646 | DNA methylase | 30 | 2 | -2.81 | 2.51E-03 |
| COG2326 | Uncharacterized conserved protein | 37 | 5 | -2.82 | 2.42E-03 |
| COG3842 | ABC-type spermidine/putrescine transport systems, ATPase components | 170 | 57 | -2.83 | 2.30E-03 |

**Table S4.** continued

| COG ID | Name | 40 °C gene count | 55 °C gene count | Z-LOR | *p* value |
| --- | --- | --- | --- | --- | --- |
| COG0725 | ABC-type molybdate transport system, periplasmic component | 43 | 7 | -2.84 | 2.28E-03 |
| COG0384 | Predicted epimerase, PhzC/PhzF homolog | 46 | 8 | -2.85 | 2.21E-03 |
| COG2204 | Response regulator containing CheY-like receiver, AAA-type ATPase, and DNA-binding domains | 224 | 80 | -2.85 | 2.21E-03 |
| COG3940 | Predicted beta-xylosidase | 46 | 8 | -2.85 | 2.21E-03 |
| COG4277 | Predicted DNA-binding protein with the Helix-hairpin-helix motif | 87 | 23 | -2.86 | 2.10E-03 |
| COG1307 | Uncharacterized protein conserved in bacteria | 206 | 72 | -2.87 | 2.08E-03 |
| COG0229 | Conserved domain frequently associated with peptide methionine sulfoxide reductase | 33 | 3 | -2.88 | 1.97E-03 |
| COG2317 | Zn-dependent carboxypeptidase | 55 | 11 | -2.88 | 2.00E-03 |
| COG4221 | Short-chain alcohol dehydrogenase of unknown specificity | 116 | 34 | -2.91 | 1.79E-03 |
| COG3538 | Uncharacterized conserved protein | 33 | 2 | -2.94 | 1.62E-03 |
| COG1101 | ABC-type uncharacterized transport system, ATPase component | 86 | 22 | -2.95 | 1.60E-03 |
| COG1134 | ABC-type polysaccharide/polyol phosphate transport system, ATPase component | 62 | 13 | -2.96 | 1.53E-03 |
| COG0282 | Acetate kinase | 142 | 44 | -2.97 | 1.48E-03 |
| COG1472 | Beta-glucosidase-related glycosidases | 287 | 106 | -2.97 | 1.51E-03 |
| COG2996 | Uncharacterized protein conserved in bacteria | 51 | 9 | -2.97 | 1.47E-03 |
| COG3158 | K+ transporter | 40 | 1 | -2.99 | 1.38E-03 |
| COG1696 | Predicted membrane protein involved in D-alanine export | 60 | 12 | -3.01 | 1.33E-03 |
| COG4198 | Uncharacterized conserved protein | 49 | 8 | -3.02 | 1.25E-03 |
| COG0859 | ADP-heptose:LPS heptosyltransferase | 38 | 4 | -3.03 | 1.23E-03 |
| COG2723 | Beta-glucosidase/6-phospho-beta-glucosidase/beta-galactosidase | 314 | 117 | -3.03 | 1.22E-03 |
| COG0469 | Pyruvate kinase | 144 | 44 | -3.06 | 1.12E-03 |
| COG4775 | Outer membrane protein/protective antigen OMA87 | 43 | 1 | -3.07 | 1.08E-03 |
| COG3384 | Uncharacterized conserved protein | 39 | 4 | -3.08 | 1.03E-03 |
| COG5438 | Predicted multitransmembrane protein | 39 | 4 | -3.08 | 1.03E-03 |
| COG4716 | Myosin-crossreactive antigen | 53 | 9 | -3.09 | 1.00E-03 |
| COG2183 | Transcriptional accessory protein | 172 | 55 | -3.11 | 9.47E-04 |
| COG1113 | Gamma-aminobutyrate permease and related permeases | 100 | 26 | -3.13 | 8.87E-04 |
| COG1609 | Transcriptional regulators | 768 | 323 | -3.13 | 8.80E-04 |

**Table S4.** continued

| COG ID | Name | 40 °C gene count | 55 °C gene count | Z-LOR | *p* value |
| --- | --- | --- | --- | --- | --- |
| COG0003 | Oxyanion-translocating ATPase | 68 | 14 | -3.14 | 8.46E-04 |
| COG4988 | ABC-type transport system involved in cytochrome bd biosynthesis, ATPase and permease components | 51 | 8 | -3.14 | 8.50E-04 |
| COG2213 | Phosphotransferase system, mannitol-specific IIBC component | 57 | 10 | -3.15 | 8.05E-04 |
| COG2368 | Aromatic ring hydroxylase | 38 | 2 | -3.15 | 8.16E-04 |
| COG0493 | NADPH-dependent glutamate synthase beta chain and related oxidoreductases | 245 | 85 | -3.18 | 7.49E-04 |
| COG3681 | Uncharacterized conserved protein | 39 | 3 | -3.18 | 7.34E-04 |
| COG1529 | Aerobic-type carbon monoxide dehydrogenase, large subunit CoxL/CutL homologs | 170 | 53 | -3.22 | 6.41E-04 |
| COG1788 | Acyl CoA:acetate/3-ketoacid CoA transferase, alpha subunit | 47 | 6 | -3.23 | 6.24E-04 |
| COG2230 | Cyclopropane fatty acid synthase and related methyltransferases | 40 | 3 | -3.23 | 6.27E-04 |
| COG3292 | Predicted periplasmic ligand-binding sensor domain | 41 | 2 | -3.26 | 5.55E-04 |
| COG2816 | NTP pyrophosphohydrolases containing a Zn-finger, probably nucleic-acid-binding | 41 | 3 | -3.27 | 5.37E-04 |
| COG0825 | Acetyl-CoA carboxylase alpha subunit | 68 | 13 | -3.29 | 5.03E-04 |
| COG0525 | Valyl-tRNA synthetase | 270 | 94 | -3.31 | 4.69E-04 |
| COG1272 | Predicted membrane protein, hemolysin III homolog | 42 | 3 | -3.31 | 4.61E-04 |
| COG1897 | Homoserine trans-succinylase | 74 | 15 | -3.31 | 4.68E-04 |
| COG0397 | Uncharacterized conserved protein | 49 | 6 | -3.33 | 4.32E-04 |
| COG1168 | Bifunctional PLP-dependent enzyme with beta-cystathionase and maltose regulon repressor activities | 120 | 32 | -3.33 | 4.32E-04 |
| COG0722 | 3-deoxy-D-arabino-heptulosonate 7-phosphate (DAHP) synthase | 56 | 1 | -3.34 | 4.24E-04 |
| COG1680 | Beta-lactamase class C and other penicillin binding proteins | 102 | 25 | -3.35 | 4.07E-04 |
| COG0574 | Phosphoenolpyruvate synthase/pyruvate phosphate dikinase | 215 | 70 | -3.37 | 3.81E-04 |
| COG1167 | Transcriptional regulators containing a DNA-binding HTH domain and an aminotransferase domain (MocR family) and their eukaryotic orthologs | 219 | 71 | -3.42 | 3.09E-04 |
| COG0426 | Uncharacterized flavoproteins | 163 | 48 | -3.43 | 2.99E-04 |
| COG1932 | Phosphoserine aminotransferase | 82 | 17 | -3.43 | 3.00E-04 |
| COG3855 | Uncharacterized protein conserved in bacteria | 62 | 1 | -3.44 | 2.90E-04 |

**Table S4.** continued

| COG ID | Name | 40 °C gene count | 55 °C gene count | Z-LOR | *p* value |
| --- | --- | --- | --- | --- | --- |
| COG0188 | Type IIA topoisomerase (DNA gyrase/topo II, topoisomerase IV), A subunit | 365 | 133 | -3.46 | 2.67E-04 |
| COG2060 | K+-transporting ATPase, A chain | 49 | 5 | -3.46 | 2.72E-04 |
| COG0024 | Methionine aminopeptidase | 139 | 38 | -3.48 | 2.46E-04 |
| COG0101 | Pseudouridylate synthase | 124 | 32 | -3.51 | 2.26E-04 |
| COG5013 | Nitrate reductase alpha subunit | 48 | 4 | -3.51 | 2.25E-04 |
| COG0652 | Peptidyl-prolyl cis-trans isomerase (rotamase) - cyclophilin family | 67 | 11 | -3.53 | 2.09E-04 |
| COG1155 | Archaeal/vacuolar-type H+-ATPase subunit A | 168 | 49 | -3.53 | 2.07E-04 |
| COG0480 | Translation elongation factors (GTPases) | 386 | 141 | -3.54 | 2.00E-04 |
| COG0339 | Zn-dependent oligopeptidases | 50 | 2 | -3.55 | 1.93E-04 |
| COG4214 | ABC-type xylose transport system, permease component | 133 | 35 | -3.56 | 1.86E-04 |
| COG1523 | Type II secretory pathway, pullulanase PulA and related glycosidases | 75 | 13 | -3.64 | 1.36E-04 |
| COG0550 | Topoisomerase IA | 317 | 109 | -3.68 | 1.16E-04 |
| COG3051 | Citrate lyase, alpha subunit | 54 | 5 | -3.68 | 1.16E-04 |
| COG3325 | Chitinase | 55 | 2 | -3.69 | 1.13E-04 |
| COG0523 | Putative GTPases (G3E family) | 62 | 8 | -3.7 | 1.09E-04 |
| COG1115 | Na+/alanine symporter | 139 | 36 | -3.7 | 1.08E-04 |
| COG0444 | ABC-type dipeptide/oligopeptide/nickel transport system, ATPase component | 374 | 133 | -3.72 | 1.01E-04 |
| COG0790 | FOG: TPR repeat, SEL1 subfamily | 58 | 2 | -3.77 | 8.30E-05 |
| COG0386 | Glutathione peroxidase | 59 | 6 | -3.8 | 7.34E-05 |
| COG4806 | L-rhamnose isomerase | 67 | 9 | -3.8 | 7.28E-05 |
| COG4603 | ABC-type uncharacterized transport system, permease component | 107 | 23 | -3.82 | 6.62E-05 |
| COG0620 | Methionine synthase II (cobalamin-independent) | 60 | 6 | -3.84 | 6.18E-05 |
| COG1733 | Predicted transcriptional regulators | 85 | 15 | -3.84 | 6.13E-05 |
| COG0855 | Polyphosphate kinase | 80 | 13 | -3.87 | 5.38E-05 |
| COG3696 | Putative silver efflux pump | 80 | 13 | -3.87 | 5.38E-05 |
| COG0443 | Molecular chaperone | 195 | 56 | -3.88 | 5.15E-05 |
| COG1026 | Predicted Zn-dependent peptidases, insulinase-like | 83 | 14 | -3.88 | 5.24E-05 |
| COG2216 | High-affinity K+ transport system, ATPase chain B | 73 | 10 | -3.94 | 4.05E-05 |

**Table S4.** continued

| COG ID | Name | 40 °C gene count | 55 °C gene count | Z-LOR | *p* value |
| --- | --- | --- | --- | --- | --- |
| COG1171 | Threonine dehydratase | 110 | 23 | -3.95 | 3.88E-05 |
| COG1629 | Outer membrane receptor proteins, mostly Fe transport | 111 | 1 | -4.03 | 2.75E-05 |
| COG0028 | Thiamine pyrophosphate-requiring enzymes [acetolactate synthase, pyruvate dehydrogenase (cytochrome), glyoxylate carboligase, phosphonopyruvate decarboxylase] | 255 | 78 | -4.06 | 2.42E-05 |
| COG0534 | Na+-driven multidrug efflux pump | 372 | 126 | -4.11 | 1.96E-05 |
| COG1554 | Trehalose and maltose hydrolases (possible phosphorylases) | 139 | 32 | -4.13 | 1.81E-05 |
| COG4586 | ABC-type uncharacterized transport system, ATPase component | 72 | 8 | -4.13 | 1.83E-05 |
| COG1052 | Lactate dehydrogenase and related dehydrogenases | 195 | 53 | -4.16 | 1.62E-05 |
| COG1904 | Glucuronate isomerase | 110 | 21 | -4.19 | 1.41E-05 |
| COG0187 | Type IIA topoisomerase (DNA gyrase/topo II, topoisomerase IV), B subunit | 348 | 114 | -4.24 | 1.13E-05 |
| COG0765 | ABC-type amino acid transport system, permease component | 263 | 79 | -4.24 | 1.12E-05 |
| COG1538 | Outer membrane protein | 97 | 15 | -4.35 | 6.71E-06 |
| COG0841 | Cation/multidrug efflux pump | 331 | 105 | -4.37 | 6.22E-06 |
| COG3507 | Beta-xylosidase | 199 | 51 | -4.48 | 3.78E-06 |
| COG0017 | Aspartyl/asparaginyl-tRNA synthetases | 154 | 34 | -4.5 | 3.48E-06 |
| COG4771 | Outer membrane receptor for ferrienterochelin and colicins | 97 | 2 | -4.51 | 3.22E-06 |
| COG1080 | Phosphoenolpyruvate-protein kinase (PTS system EI component in bacteria) | 190 | 47 | -4.53 | 2.94E-06 |
| COG1132 | ABC-type multidrug transport system, ATPase and permease components | 1054 | 418 | -4.61 | 2.00E-06 |
| COG0562 | UDP-galactopyranose mutase | 99 | 13 | -4.65 | 1.67E-06 |
| COG0659 | Sulfate permease and related transporters (MFS superfamily) | 85 | 7 | -4.67 | 1.48E-06 |
| COG1020 | Non-ribosomal peptide synthetase modules and related proteins | 89 | 9 | -4.67 | 1.53E-06 |
| COG0642 | Signal transduction histidine kinase | 589 | 209 | -4.69 | 1.37E-06 |
| COG4977 | Transcriptional regulator containing an amidase domain and an AraC-type DNA-binding HTH domain | 151 | 31 | -4.69 | 1.38E-06 |
| COG3537 | Putative alpha-1,2-mannosidase | 87 | 4 | -4.73 | 1.10E-06 |
| COG4206 | Outer membrane cobalamin receptor protein | 93 | 3 | -4.73 | 1.12E-06 |
| COG1640 | 4-alpha-glucanotransferase | 91 | 9 | -4.74 | 1.09E-06 |

**Table S4.** continued

| COG ID | Name | 40 °C gene count | 55 °C gene count | Z-LOR | *p* value |
| --- | --- | --- | --- | --- | --- |
| COG4225 | Predicted unsaturated glucuronyl hydrolase involved in regulation of bacterial surface properties, and related proteins | 99 | 12 | -4.75 | 1.03E-06 |
| COG3845 | ABC-type uncharacterized transport systems, ATPase components | 229 | 59 | -4.78 | 8.90E-07 |
| COG4753 | Response regulator containing CheY-like receiver domain and AraC-type DNA-binding domain | 520 | 177 | -4.82 | 7.30E-07 |
| COG0474 | Cation transport ATPase | 565 | 193 | -4.99 | 3.09E-07 |
| COG0178 | Excinuclease ATPase subunit | 398 | 119 | -5.25 | 7.50E-08 |
| COG1126 | ABC-type polar amino acid transport system, ATPase component | 351 | 100 | -5.27 | 6.93E-08 |
| COG0296 | 1,4-alpha-glucan branching enzyme | 141 | 21 | -5.32 | 5.05E-08 |
| COG2200 | FOG: EAL domain | 264 | 64 | -5.44 | 2.61E-08 |
| COG0448 | ADP-glucose pyrophosphorylase | 150 | 22 | -5.52 | 1.67E-08 |
| COG0514 | Superfamily II DNA helicase | 140 | 18 | -5.56 | 1.34E-08 |
| COG3459 | Cellobiose phosphorylase | 286 | 70 | -5.62 | 9.76E-09 |
| COG1136 | ABC-type antimicrobial peptide transport system, ATPase component | 534 | 165 | -5.79 | 3.43E-09 |
| COG2274 | ABC-type bacteriocin/lantibiotic exporters, contain an N-terminal double-glycine peptidase domain | 312 | 74 | -6.04 | 7.91E-10 |
| COG2207 | AraC-type DNA-binding domain-containing proteins | 483 | 139 | -6.1 | 5.29E-10 |
| COG0058 | Glucan phosphorylase | 230 | 40 | -6.37 | 9.66E-11 |
| COG0513 | Superfamily II DNA and RNA helicases | 281 | 57 | -6.45 | 5.63E-11 |
| COG1129 | ABC-type sugar transport system, ATPase component | 650 | 198 | -6.54 | 3.16E-11 |
| COG1882 | Pyruvate-formate lyase | 290 | 55 | -6.83 | 4.32E-12 |
| COG0745 | Response regulators consisting of a CheY-like receiver domain and a winged-helix DNA-binding domain | 937 | 309 | -6.88 | 2.96E-12 |
| COG0583 | Transcriptional regulator | 436 | 105 | -7.04 | 9.53E-13 |
| COG2205 | Osmosensitive K+ channel histidine kinase | 284 | 48 | -7.17 | 3.67E-13 |
| COG0488 | ATPase components of ABC transporters with duplicated ATPase domains | 608 | 100 | -10.64 | 0.00E+00 |

^1^ Positive Z-LOR scores indicate COGs enriched in the thermophilic metagenome and negative Z-LOR scores indicate COGs enriched in the mesophilic metagenome. Significance was evaluated following false discovery rate correction where *p* ≤ 4.55^-3^ was considered to be significant.

^2^ Note: Although raw gene counts are provided here, all statistics were generated using gene category proportions (i.e., relative abundances).
