# Supplementary material for: Mesophilic and Thermophilic Conditions Select for Unique but Highly Parallel Microbial Communities to Perform Carboxylate Platform Biomass Conversion
Source: PLoS One. 2012 Jun 22;7(6):e39689. doi: 10.1371/journal.pone.0039689 (PMC3382152; doi:10.1371/journal.pone.0039689)
Supplement: Table S5 — Enzymes significantly enriched or depleted between the thermophilic and mesophilic metagenomes, as determined by z -normalized log odds ratios (Z-LOR). (DOC) [file pone.0039689.s007.doc]

**Table S5.** Enzymes significantly enriched or depleted between the thermophilic and mesophilic metagenomes, as determined by *z*-normalized log odds ratios (Z-LOR).^1^

| EC number | Name | 40 °C gene count ^2^ | 55 °C gene count | Z-LOR | *p* value |
| --- | --- | --- | --- | --- | --- |
| EC:2.7.1.69 | Protein-N(pi)-phosphohistidine--sugar phosphotransferase. | 219 | 413 | 15.45 | 0.00E+00 |
| EC:3.5.1.28 | N-acetylmuramoyl-L-alanine amidase. | 257 | 323 | 10.57 | 0.00E+00 |
| EC:1.3.1.74 | 2-alkenal reductase. | 56 | 118 | 8.62 | 0.00E+00 |
| EC:5.4.99.2 | Methylmalonyl-CoA mutase. | 49 | 97 | 7.63 | 1.20E-14 |
| EC:2.7.7.13 | Mannose-1-phosphate guanylyltransferase. | 14 | 65 | 7.43 | 5.43E-14 |
| EC:2.1.1.72 | Site-specific DNA-methyltransferase (adenine-specific). | 426 | 369 | 7.19 | 3.35E-13 |
| EC:2.7.8.6 | Undecaprenyl-phosphate galactose phosphotransferase. | 6 | 50 | 6.42 | 6.78E-11 |
| EC:1.8.98.1 | CoB--CoM heterodisulfide reductase. | 11 | 48 | 6.36 | 9.92E-11 |
| EC:6.2.1.3 | Long-chain-fatty-acid--CoA ligase. | 109 | 127 | 6.18 | 3.26E-10 |
| EC:1.3.99.3 | Acyl-CoA dehydrogenase. | 5 | 46 | 6.1 | 5.27E-10 |
| EC:3.5.2.14 | N-methylhydantoinase (ATP-hydrolyzing). | 19 | 50 | 6.02 | 8.99E-10 |
| EC:3.4.21.- | Hydrolases. Acting on peptide bonds (peptide hydrolases). Serine endopeptidases. | 150 | 155 | 5.99 | 1.02E-09 |
| EC:1.2.7.5 | Aldehyde ferredoxin oxidoreductase. | 38 | 66 | 5.92 | 1.61E-09 |
| EC:6.2.1.- | Ligases. Forming carbon-sulfur bonds. Acid--thiol ligases. | 55 | 78 | 5.7 | 6.14E-09 |
| EC:5.4.2.2 | Phosphoglucomutase. | 29 | 55 | 5.64 | 8.69E-09 |
| EC:4.1.1.18 | Lysine decarboxylase. | 62 | 83 | 5.63 | 8.95E-09 |
| EC:3.2.1.4 | Cellulase. | 90 | 104 | 5.54 | 1.48E-08 |
| EC:6.3.4.14 | Biotin carboxylase. | 14 | 39 | 5.39 | 3.61E-08 |
| EC:2.7.11.1 | Non-specific serine/threonine protein kinase. | 179 | 166 | 5.37 | 3.99E-08 |
| EC:3.5.1.14 | Aminoacylase. | 37 | 59 | 5.34 | 4.62E-08 |
| EC:3.5.4.25 | GTP cyclohydrolase II. | 8 | 34 | 5.34 | 4.52E-08 |
| EC:1.2.7.1 | Pyruvate synthase. | 68 | 84 | 5.3 | 5.75E-08 |
| EC:2.4.1.187 | N-acetylglucosaminyldiphosphoundecaprenol N-acetyl-beta-D- mannosaminyltransferase. | 16 | 39 | 5.2 | 9.87E-08 |
| EC:1.9.3.1 | Cytochrome-c oxidase. | 49 | 67 | 5.14 | 1.37E-07 |
| EC:3.1.11.5 | Exodeoxyribonuclease V. | 211 | 183 | 5.06 | 2.07E-07 |
| EC:4.2.1.17 | Enoyl-CoA hydratase. | 76 | 87 | 5.02 | 2.54E-07 |
| EC:3.6.4.6 | Vesicle-fusing ATPase. | 3 | 31 | 4.94 | 3.84E-07 |
| EC:1.2.7.3 | 2-oxoglutarate synthase. | 220 | 186 | 4.88 | 5.36E-07 |
| EC:3.2.1.91 | Cellulose 1,4-beta-cellobiosidase. | 16 | 36 | 4.87 | 5.49E-07 |

**Table S5.** continued

| EC number | Name | 40 °C gene count | 55 °C gene count | Z-LOR | *p* value |
| --- | --- | --- | --- | --- | --- |
| EC:6.5.1.- | Ligases. Forming phosphoric ester bonds. Ligases that form phosphoric-ester bonds. | 4 | 27 | 4.78 | 8.61E-07 |
| EC:1.21.4.2 | Glycine reductase. | 38 | 54 | 4.75 | 1.04E-06 |
| EC:3.4.23.- | Hydrolases. Acting on peptide bonds (peptide hydrolases). Aspartic endopeptidases. | 11 | 30 | 4.7 | 1.30E-06 |
| EC:2.7.1.2 | Glucokinase. | 96 | 98 | 4.69 | 1.34E-06 |
| EC:3.4.13.9 | Xaa-Pro dipeptidase. | 60 | 71 | 4.69 | 1.39E-06 |
| EC:3.1.3.71 | 2-phosphosulfolactate phosphatase. | 9 | 28 | 4.67 | 1.53E-06 |
| EC:2.5.1.29 | Farnesyltranstransferase. | 4 | 24 | 4.53 | 2.99E-06 |
| EC:4.1.1.37 | Uroporphyrinogen decarboxylase. | 25 | 41 | 4.52 | 3.03E-06 |
| EC:6.1.1.24 | Glutamate--tRNA(Gln) ligase. | 25 | 41 | 4.52 | 3.03E-06 |
| EC:2.7.1.31 | Glycerate kinase. | 54 | 64 | 4.46 | 4.19E-06 |
| EC:2.7.1.144 | Tagatose-6-phosphate kinase. | 16 | 32 | 4.4 | 5.48E-06 |
| EC:1.2.1.2 | Formate dehydrogenase. | 121 | 112 | 4.39 | 5.56E-06 |
| EC:1.1.1.14 | L-iditol 2-dehydrogenase. | 40 | 52 | 4.35 | 6.69E-06 |
| EC:6.2.1.1 | Acetate--CoA ligase. | 55 | 63 | 4.28 | 9.51E-06 |
| EC:3.2.- | Hydrolases. Glycosylases. | 78 | 80 | 4.27 | 9.99E-06 |
| EC:1.2.4.4 | 3-methyl-2-oxobutanoate dehydrogenase (2-methylpropanoyl-transferring). | 29 | 42 | 4.24 | 1.12E-05 |
| EC:2.5.1.30 | Trans-hexaprenyltranstransferase. | 34 | 46 | 4.22 | 1.19E-05 |
| EC:3.1.21.5 | Type III site-specific deoxyribonuclease. | 72 | 75 | 4.21 | 1.29E-05 |
| EC:2.7.1.12 | Gluconokinase. | 27 | 40 | 4.2 | 1.33E-05 |
| EC:1.2.4.1 | Pyruvate dehydrogenase (acetyl-transferring). | 102 | 96 | 4.17 | 1.53E-05 |
| EC:2.1.1.148 | Thymidylate synthase (FAD). | 17 | 31 | 4.16 | 1.63E-05 |
| EC:4.2.1.30 | Glycerol dehydratase. | 25 | 38 | 4.16 | 1.57E-05 |
| EC:1.10.2.- | Oxidoreductases. Acting on diphenols and related substances as donors. With a cytochrome as acceptor. | 4 | 20 | 4.13 | 1.81E-05 |
| EC:1.4.7.1 | Glutamate synthase (ferredoxin). | 22 | 35 | 4.11 | 2.00E-05 |
| EC:3.1.1.41 | Cephalosporin-C deacetylase. | 13 | 27 | 4.1 | 2.07E-05 |
| EC:1.12.5.1 | Hydrogen:quinone oxidoreductase. | 2 | 21 | 4.06 | 2.45E-05 |
| EC:3.1.3.16 | Phosphoprotein phosphatase. | 114 | 103 | 4.06 | 2.45E-05 |
| EC:4.1.1.19 | Arginine decarboxylase. | 48 | 55 | 4 | 3.21E-05 |

**Table S5.** continued

| EC number | Name | 40 °C gene count | 55 °C gene count | Z-LOR | *p* value |
| --- | --- | --- | --- | --- | --- |
| EC:2.3.3.9 | Malate synthase. | 15 | 28 | 3.99 | 3.28E-05 |
| EC:2.3.1.31 | Homoserine O-acetyltransferase. | 11 | 24 | 3.94 | 4.13E-05 |
| EC:3.4.19.1 | Acylaminoacyl-peptidase. | 8 | 21 | 3.89 | 4.91E-05 |
| EC:1.4.1.9 | Leucine dehydrogenase. | 7 | 20 | 3.88 | 5.27E-05 |
| EC:1.13.12.16 | Nitronate monooxygenase. | 6 | 19 | 3.86 | 5.76E-05 |
| EC:3.5.4.28 | S-adenosylhomocysteine deaminase. | 6 | 19 | 3.86 | 5.76E-05 |
| EC:2.7.7.19 | Polynucleotide adenylyltransferase. | 38 | 46 | 3.85 | 5.87E-05 |
| EC:2.7.7.56 | tRNA nucleotidyltransferase. | 22 | 33 | 3.85 | 6.00E-05 |
| EC:4.1.2.40 | Tagatose-bisphosphate aldolase. | 27 | 37 | 3.83 | 6.52E-05 |
| EC:3.4.11.- | Hydrolases. Acting on peptide bonds (peptide hydrolases). Aminopeptidases. | 154 | 126 | 3.77 | 8.19E-05 |
| EC:2.3.3.14 | Homocitrate synthase. | 1 | 24 | 3.75 | 8.70E-05 |
| EC:2.4.2.28 | S-methyl-5'-thioadenosine phosphorylase. | 8 | 20 | 3.75 | 8.78E-05 |
| EC:3.1.2.1 | Acetyl-CoA hydrolase. | 8 | 20 | 3.75 | 8.78E-05 |
| EC:2.1.1.113 | Site-specific DNA-methyltransferase (cytosine-N(4)-specific). | 2 | 17 | 3.74 | 9.33E-05 |
| EC:2.7.1.25 | Adenylyl-sulfate kinase. | 7 | 19 | 3.74 | 9.36E-05 |
| EC:2.7.7.21 | tRNA cytidylyltransferase. | 38 | 45 | 3.73 | 9.48E-05 |
| EC:2.7.7.25 | tRNA adenylyltransferase. | 38 | 45 | 3.73 | 9.48E-05 |
| EC:3.1.4.- | Hydrolases. Acting on ester bonds. Phosphoric diester hydrolases. | 38 | 45 | 3.73 | 9.48E-05 |
| EC:3.4.13.19 | Membrane dipeptidase. | 37 | 44 | 3.71 | 1.06E-04 |
| EC:2.7.1.59 | N-acetylglucosamine kinase. | 3 | 16 | 3.7 | 1.08E-04 |
| EC:3.1.3.41 | 4-nitrophenylphosphatase. | 5 | 17 | 3.69 | 1.12E-04 |
| EC:3.4.23.43 | Prepilin peptidase. | 34 | 41 | 3.62 | 1.46E-04 |
| EC:2.3.1.12 | Dihydrolipoyllysine-residue acetyltransferase. | 56 | 57 | 3.57 | 1.81E-04 |
| EC:5.3.1.23 | S-methyl-5-thioribose-1-phosphate isomerase. | 22 | 31 | 3.57 | 1.76E-04 |
| EC:3.5.3.11 | Agmatinase. | 36 | 42 | 3.55 | 1.89E-04 |
| EC:1.1.1.37 | Malate dehydrogenase. | 26 | 34 | 3.54 | 2.02E-04 |
| EC:1.5.5.- | Oxidoreductases. Acting on the CH-NH group of donors. With a quinone or similar compound as acceptor. | 4 | 15 | 3.51 | 2.25E-04 |
| EC:3.4.- | Hydrolases. Acting on peptide bonds (peptide hydrolases). | 422 | 287 | 3.51 | 2.28E-04 |

**Table S5.** continued

| EC number | Name | 40 °C gene count | 55 °C gene count | Z-LOR | *p* value |
| --- | --- | --- | --- | --- | --- |
| EC:3.1.21.3 | Type I site-specific deoxyribonuclease. | 532 | 352 | 3.5 | 2.29E-04 |
| EC:2.7.1.156 | Adenosylcobinamide kinase. | 10 | 20 | 3.48 | 2.54E-04 |
| EC:2.7.7.62 | Adenosylcobinamide-phosphate guanylyltransferase. | 10 | 20 | 3.48 | 2.54E-04 |
| EC:3.4.15.6 | Cyanophycinase. | 10 | 20 | 3.48 | 2.54E-04 |
| EC:3.2.1.8 | Endo-1,4-beta-xylanase. | 33 | 39 | 3.47 | 2.62E-04 |
| EC:5.4.3.2 | Lysine 2,3-aminomutase. | 23 | 31 | 3.46 | 2.72E-04 |
| EC:3.5.2.10 | Creatininase. | 8 | 18 | 3.45 | 2.85E-04 |
| EC:1.12.2.1 | Cytochrome-c3 hydrogenase. | 2 | 14 | 3.44 | 2.93E-04 |
| EC:2.7.7.9 | UTP--glucose-1-phosphate uridylyltransferase. | 59 | 58 | 3.44 | 2.90E-04 |
| EC:1.2.1.3 | Aldehyde dehydrogenase (NAD(+)). | 75 | 69 | 3.42 | 3.17E-04 |
| EC:1.4.1.1 | Alanine dehydrogenase. | 20 | 28 | 3.38 | 3.62E-04 |
| EC:6.3.3.2 | 5-formyltetrahydrofolate cyclo-ligase. | 20 | 28 | 3.38 | 3.62E-04 |
| EC:2.7.10.1 | Receptor protein-tyrosine kinase. | 4 | 14 | 3.36 | 3.87E-04 |
| EC:2.7.4.6 | Nucleoside-diphosphate kinase. | 16 | 24 | 3.28 | 5.19E-04 |
| EC:6.4.1.3 | Propionyl-CoA carboxylase. | 104 | 87 | 3.27 | 5.41E-04 |
| EC:3.4.25.- | Hydrolases. Acting on peptide bonds (peptide hydrolases). Threonine endopeptidases. | 15 | 23 | 3.26 | 5.65E-04 |
| EC:3.1.6.1 | Arylsulfatase. | 1 | 15 | 3.25 | 5.68E-04 |
| EC:3.1.4.16 | 2',3'-cyclic-nucleotide 2'-phosphodiesterase. | 30 | 35 | 3.24 | 5.88E-04 |
| EC:4.1.2.17 | L-fuculose-phosphate aldolase. | 34 | 38 | 3.24 | 6.02E-04 |
| EC:1.2.7.7 | 3-methyl-2-oxobutanoate dehydrogenase (ferredoxin). | 2 | 12 | 3.2 | 6.85E-04 |
| EC:2.7.- | Transferases. Transferring phosphorous-containing groups. | 18 | 25 | 3.18 | 7.48E-04 |
| EC:6.4.1.6 | Acetone carboxylase. | 1 | 14 | 3.18 | 7.35E-04 |
| EC:2.7.1.60 | N-acylmannosamine kinase. | 3 | 12 | 3.16 | 7.91E-04 |
| EC:1.17.1.4 | Xanthine dehydrogenase. | 70 | 63 | 3.15 | 8.03E-04 |
| EC:6.3.1.5 | NAD(+) synthase. | 17 | 24 | 3.15 | 8.22E-04 |
| EC:2.3.1.29 | Glycine C-acetyltransferase. | 31 | 35 | 3.14 | 8.44E-04 |
| EC:1.1.1.35 | 3-hydroxyacyl-CoA dehydrogenase. | 46 | 46 | 3.13 | 8.67E-04 |
| EC:1.1.1.47 | Glucose 1-dehydrogenase. | 9 | 17 | 3.13 | 8.83E-04 |
| EC:2.4.1.83 | Dolichyl-phosphate beta-D-mannosyltransferase. | 26 | 31 | 3.12 | 9.12E-04 |
| EC:1.1.1.53 | 3-alpha-(or 20-beta)-hydroxysteroid dehydrogenase. | 1 | 13 | 3.1 | 9.64E-04 |
| EC:1.8.99.3 | Hydrogensulfite reductase. | 2 | 11 | 3.07 | 1.08E-03 |

**Table S5.** continued

| EC number | Name | 40 °C gene count | 55 °C gene count | Z-LOR | *p* value |
| --- | --- | --- | --- | --- | --- |
| EC:5.3.2.- | Isomerases. Intramolecular oxidoreductases. Interconverting keto- and enol- groups. | 2 | 11 | 3.07 | 1.08E-03 |
| EC:6.2.1.26 | o-succinylbenzoate--CoA ligase. | 14 | 21 | 3.07 | 1.08E-03 |
| EC:3.2.1.67 | Galacturan 1,4-alpha-galacturonidase. | 5 | 13 | 3.06 | 1.12E-03 |
| EC:3.5.1.4 | Amidase. | 36 | 38 | 3.04 | 1.18E-03 |
| EC:1.9.3.- | Oxidoreductases. Acting on a heme group of donors. With oxygen as acceptor. | 23 | 28 | 3.02 | 1.26E-03 |
| EC:3.5.4.- | Hydrolases. Acting on carbon-nitrogen bonds, other than peptide bonds. In cyclic amidines. | 12 | 19 | 3.02 | 1.27E-03 |
| EC:2.5.1.3 | Thiamine-phosphate diphosphorylase. | 39 | 40 | 3.01 | 1.29E-03 |
| EC:3.4.24.84 | Ste24 endopeptidase. | 1 | 12 | 3.01 | 1.29E-03 |
| EC:2.3.1.16 | Acetyl-CoA C-acyltransferase. | 31 | 34 | 3 | 1.34E-03 |
| EC:3.4.19.11 | Gamma-D-glutamyl-meso-diaminopimelate peptidase. | 22 | 27 | 2.99 | 1.41E-03 |
| EC:3.2.1.37 | Xylan 1,4-beta-xylosidase. | 67 | 59 | 2.95 | 1.61E-03 |
| EC:1.14.14.3 | Alkanal monooxygenase (FMN-linked). | 8 | 15 | 2.93 | 1.71E-03 |
| EC:2.1.1.71 | Phosphatidyl-N-methylethanolamine N-methyltransferase. | 2 | 10 | 2.92 | 1.75E-03 |
| EC:2.7.4.7 | Phosphomethylpyrimidine kinase. | 60 | 54 | 2.92 | 1.75E-03 |
| EC:3.4.21.62 | Subtilisin. | 2 | 10 | 2.92 | 1.75E-03 |
| EC:4.- | Lyases. | 63 | 56 | 2.92 | 1.78E-03 |
| EC:2.7.2.1 | Acetate kinase. | 140 | 44 | -2.92 | 1.74E-03 |
| EC:6.1.1.9 | Valine--tRNA ligase. | 259 | 95 | -2.92 | 1.74E-03 |
| EC:2.3.1.79 | Maltose O-acetyltransferase. | 36 | 4 | -2.93 | 1.69E-03 |
| EC:6.1.1.3 | Threonine--tRNA ligase. | 202 | 70 | -2.94 | 1.66E-03 |
| EC:1.4.3.4 | Monoamine oxidase. | 33 | 2 | -2.95 | 1.57E-03 |
| EC:2.1.1.79 | Cyclopropane-fatty-acyl-phospholipid synthase. | 38 | 1 | -2.95 | 1.61E-03 |
| EC:3.1.3.11 | Fructose-bisphosphatase. | 83 | 21 | -2.95 | 1.57E-03 |
| EC:1.5.1.7 | Saccharopine dehydrogenase (NAD(+), L-lysine-forming). | 51 | 9 | -2.99 | 1.38E-03 |
| EC:5.2.1.8 | Peptidylprolyl isomerase. | 168 | 55 | -2.99 | 1.41E-03 |
| EC:1.16.3.1 | Ferroxidase. | 35 | 3 | -3 | 1.35E-03 |
| EC:5.99.1.- | Isomerases. Other isomerases. Sole sub-subclass for isomerases that do not belong in the other subclasses. | 154 | 49 | -3 | 1.33E-03 |
| EC:4.1.2.19 | Rhamnulose-1-phosphate aldolase. | 38 | 4 | -3.04 | 1.18E-03 |

**Table S5.** continued

| EC number | Name | 40 °C gene count | 55 °C gene count | Z-LOR | *p* value |
| --- | --- | --- | --- | --- | --- |
| EC:3.2.1.22 | Alpha-galactosidase. | 226 | 79 | -3.05 | 1.14E-03 |
| EC:3.1.26.11 | Ribonuclease Z. | 69 | 15 | -3.07 | 1.08E-03 |
| EC:3.2.1.45 | Glucosylceramidase. | 58 | 11 | -3.07 | 1.07E-03 |
| EC:1.17.4.2 | Ribonucleoside-triphosphate reductase. | 290 | 106 | -3.12 | 9.05E-04 |
| EC:2.2.1.6 | Acetolactate synthase. | 277 | 100 | -3.14 | 8.40E-04 |
| EC:1.11.1.6 | Catalase. | 68 | 14 | -3.16 | 7.85E-04 |
| EC:6.1.1.21 | Histidine--tRNA ligase. | 143 | 43 | -3.16 | 7.96E-04 |
| EC:2.3.1.46 | Homoserine O-succinyltransferase. | 71 | 15 | -3.17 | 7.51E-04 |
| EC:3.1.3.18 | Phosphoglycolate phosphatase. | 111 | 30 | -3.19 | 7.19E-04 |
| EC:1.1.1.284 | S-(hydroxymethyl)glutathione dehydrogenase. | 44 | 5 | -3.23 | 6.29E-04 |
| EC:6.1.1.5 | Isoleucine--tRNA ligase. | 290 | 104 | -3.27 | 5.47E-04 |
| EC:3.6.1.22 | NAD(+) diphosphatase. | 41 | 3 | -3.28 | 5.17E-04 |
| EC:3.1.1.53 | Sialate O-acetylesterase. | 65 | 12 | -3.3 | 4.83E-04 |
| EC:1.14.13.3 | 4-hydroxyphenylacetate 3-monooxygenase. | 43 | 2 | -3.34 | 4.20E-04 |
| EC:4.3.1.19 | Threonine ammonia-lyase. | 99 | 24 | -3.36 | 3.89E-04 |
| EC:4.3.1.15 | Diaminopropionate ammonia-lyase. | 45 | 3 | -3.45 | 2.83E-04 |
| EC:5.3.1.14 | L-rhamnose isomerase. | 82 | 17 | -3.46 | 2.74E-04 |
| EC:6.4.1.2 | Acetyl-CoA carboxylase. | 241 | 80 | -3.49 | 2.41E-04 |
| EC:1.8.4.11 | Peptide-methionine (S)-S-oxide reductase. | 75 | 14 | -3.52 | 2.13E-04 |
| EC:3.2.1.21 | Beta-glucosidase. | 591 | 234 | -3.55 | 1.90E-04 |
| EC:4.1.1.17 | Ornithine decarboxylase. | 49 | 4 | -3.56 | 1.83E-04 |
| EC:6.3.5.1 | NAD(+) synthase (glutamine-hydrolyzing). | 133 | 35 | -3.59 | 1.63E-04 |
| EC:2.6.1.52 | Phosphoserine transaminase. | 85 | 17 | -3.6 | 1.58E-04 |
| EC:2.7.3.9 | Phosphoenolpyruvate--protein phosphotransferase. | 168 | 48 | -3.67 | 1.22E-04 |
| EC:1.4.1.4 | Glutamate dehydrogenase (NADP(+)). | 106 | 24 | -3.68 | 1.15E-04 |
| EC:3.4.13.3 | Xaa-His dipeptidase. | 81 | 15 | -3.68 | 1.17E-04 |
| EC:1.1.1.29 | Glycerate dehydrogenase. | 52 | 3 | -3.71 | 1.06E-04 |
| EC:1.1.1.58 | Tagaturonate reductase. | 88 | 17 | -3.74 | 9.09E-05 |
| EC:1.6.5.- | Oxidoreductases. Acting on NADH or NADPH. With a quinone or similar compound as acceptor. | 55 | 5 | -3.74 | 9.32E-05 |
| EC:4.1.1.- | Lyases. Carbon-carbon lyases. Carboxy-lyases. | 165 | 46 | -3.75 | 8.91E-05 |
| EC:1.8.4.12 | Peptide-methionine (R)-S-oxide reductase. | 63 | 8 | -3.76 | 8.50E-05 |

**Table S5.** continued

| EC number | Name | 40 °C gene count | 55 °C gene count | Z-LOR | *p* value |
| --- | --- | --- | --- | --- | --- |
| EC:2.1.1.14 | 5-methyltetrahydropteroyltriglutamate--homocysteine S-methyltransferase. | 63 | 8 | -3.76 | 8.50E-05 |
| EC:1.11.1.9 | Glutathione peroxidase. | 58 | 6 | -3.77 | 8.20E-05 |
| EC:2.7.4.1 | Polyphosphate kinase. | 78 | 13 | -3.8 | 7.15E-05 |
| EC:3.6.1.- | Hydrolases. Acting on acid anhydrides. In phosphorous-containing anhydrides. | 1806 | 801 | -3.81 | 6.88E-05 |
| EC:3.6.3.2 | Magnesium-importing ATPase. | 60 | 2 | -3.82 | 6.56E-05 |
| EC:1.1.1.28 | D-lactate dehydrogenase. | 56 | 3 | -3.84 | 6.21E-05 |
| EC:3.4.11.18 | Methionyl aminopeptidase. | 151 | 39 | -3.9 | 4.72E-05 |
| EC:1.2.1.10 | Acetaldehyde dehydrogenase (acetylating). | 223 | 67 | -3.95 | 3.92E-05 |
| EC:4.1.1.3 | Oxaloacetate decarboxylase. | 259 | 81 | -4.01 | 3.07E-05 |
| EC:6.1.1.23 | Aspartate--tRNA(Asn) ligase. | 62 | 3 | -4.02 | 2.92E-05 |
| EC:4.2.1.3 | Aconitate hydratase. | 262 | 82 | -4.03 | 2.84E-05 |
| EC:5.3.1.12 | Glucuronate isomerase. | 105 | 20 | -4.12 | 1.87E-05 |
| EC:3.2.1.- | Hydrolases. Glycosylases. Glycosidases, i.e. enzymes hydrolyzing O- and S-glycosyl compounds. | 124 | 26 | -4.22 | 1.23E-05 |
| EC:3.6.3.16 | Arsenite-transporting ATPase. | 127 | 25 | -4.45 | 4.35E-06 |
| EC:5.4.99.9 | UDP-galactopyranose mutase. | 96 | 14 | -4.45 | 4.30E-06 |
| EC:4.1.3.6 | Citrate (pro-3S)-lyase. | 94 | 13 | -4.48 | 3.71E-06 |
| EC:6.1.1.18 | Glutamine--tRNA ligase. | 108 | 18 | -4.48 | 3.81E-06 |
| EC:5.99.1.2 | DNA topoisomerase. | 403 | 134 | -4.5 | 3.33E-06 |
| EC:2.1.1.37 | DNA (cytosine-5-)-methyltransferase. | 210 | 52 | -4.8 | 7.87E-07 |
| EC:2.4.1.25 | 4-alpha-glucanotransferase. | 98 | 11 | -4.83 | 6.94E-07 |
| EC:1.2.7.- | Oxidoreductases. Acting on the aldehyde or oxo group of donors. With an iron-sulfur protein as acceptor. | 466 | 155 | -4.84 | 6.41E-07 |
| EC:1.4.1.13 | Glutamate synthase (NADPH). | 519 | 175 | -4.98 | 3.15E-07 |
| EC:1.4.1.14 | Glutamate synthase (NADH). | 519 | 175 | -4.98 | 3.15E-07 |
| EC:2.7.9.2 | Pyruvate, water dikinase. | 177 | 35 | -5.24 | 8.17E-08 |
| EC:2.7.7.27 | Glucose-1-phosphate adenylyltransferase. | 142 | 22 | -5.29 | 6.02E-08 |
| EC:5.99.1.3 | DNA topoisomerase (ATP-hydrolyzing). | 619 | 209 | -5.43 | 2.83E-08 |
| EC:2.4.1.18 | 1,4-alpha-glucan branching enzyme. | 136 | 18 | -5.46 | 2.34E-08 |
| EC:3.6.3.17 | Monosaccharide-transporting ATPase. | 720 | 251 | -5.49 | 2.00E-08 |

**Table S5.** continued

| EC number | Name | 40 °C gene count | 55 °C gene count | Z-LOR | *p* value |
| --- | --- | --- | --- | --- | --- |
| EC:3.6.3.12 | Potassium-transporting ATPase. | 149 | 18 | -5.86 | 2.35E-09 |
| EC:2.4.1.1 | Phosphorylase. | 245 | 43 | -6.58 | 2.30E-11 |
| EC:2.3.1.54 | Formate C-acetyltransferase. | 341 | 58 | -7.89 | 1.55E-15 |

^1^ Positive Z-LOR scores indicate enzymes enriched in the thermophilic metagenome and negative Z-LOR scores indicate enzymes enriched in the mesophilic metagenome. Significance was evaluated following false discovery rate correction where *p* ≤ 1.78^-3^ was considered to be significant.

^2^Note: Although raw gene counts are provided here, all statistics were generated using gene category proportions (i.e., relative abundances)
