# Supplementary material for: Mesophilic and Thermophilic Conditions Select for Unique but Highly Parallel Microbial Communities to Perform Carboxylate Platform Biomass Conversion
Source: PLoS One. 2012 Jun 22;7(6):e39689. doi: 10.1371/journal.pone.0039689 (PMC3382152; doi:10.1371/journal.pone.0039689)
Supplement: Table S6 — Genomes used to identify phylogenetic order-level core genes and conserved single copy genes (CSGS). (DOC) [file pone.0039689.s008.doc]

**Table S6.** Genomes used to identify phylogenetic order-level core genes and conserved single copy genes (CSGS)

| **Clostridiales (123 core genes, 33 CSGS)** | |
| --- | --- |
| *Alkaliphilus metalliredigens QYMF* | *Clostridium cf saccharolyticum K10* |
| *Alkaliphilus oremlandii OhILAs* | *Clostridium difficile 630 epidemic type X* |
| *Anaerococcus prevotii PC 1 DSM 20548* | *Clostridium difficile CD196* |
| *Butyrivibrio fibrisolvens 16 4* | *Clostridium difficile R20291* |
| *Butyrivibrio proteoclasticus B316* | *Clostridium kluyveri DSM 555* |
| *Candidatus Desulforudis audaxviator MP104C* | *Clostridium kluyveri NBRC 12016* |
| *Clostridiales genomosp BVAB3 UPII9 5* | *Clostridium ljungdahlii PETC DSM 13528* |
| *Clostridium acetobutylicum ATCC 824* | *Clostridium novyi NT* |
| *Clostridium beijerinckii NCIMB 8052* | *Clostridium perfringens 13* |
| *Clostridium botulinum A2 Kyoto F* | *Clostridium perfringens ATCC 13124* |
| *Clostridium botulinum B Eklund 17B* | *Clostridium perfringens SM101* |
| *Clostridium botulinum Ba4 657* | *Clostridium phytofermentans ISDg* |
| *Clostridium botulinum BoNT A1 Hall* | *Clostridium saccharolyticum WM1 DSM 2544* |
| *Clostridium botulinum BoNT A1 ATCC 19397* | *Clostridium sticklandii DSM 519* |
| *Clostridium botulinum BoNT A3 Loch Maree* | *Clostridium tetani Massachusetts E88* |
| *Clostridium botulinum BoNT B1 Okra* | *Clostridium thermocellum ATCC 27405* |
| *Clostridium botulinum E3 Alaska E43* | *Clostridium thermocellum LQ8 DSM 1313* |
| *Clostridium botulinum F 230613* | *Coprococcus catus GD 7* |
| *Clostridium botulinum F Langeland* | *Desulfitobacterium hafniense DCB 2* |
| *Clostridium botulinum type A Hall* | *Desulfitobacterium hafniense Y51* |
| *Clostridium cellulolyticum H10* | *Desulfotomaculum acetoxidans 5575 DSM 771* |
| *Clostridium cellulovorans 743B ATCC 35296* | *Desulfotomaculum reducens MI 1* |

**Table S6.** continued

| **Clostridiales, continued** | |
| --- | --- |
| *Ethanoligenens harbinense YUAN 3T DSM 18485* | *Roseburia intestinalis M50 1* |
| *Eubacterium eligens ATCC 27750* | *Roseburia intestinalis XB6B4* |
| *Eubacterium limosum KIST612* | *Ruminococcus albus 7* |
| *Eubacterium rectale ATCC 33656* | *Ruminococcus bromii L2 63* |
| *Eubacterium rectale DSM 17629* | *Ruminococcus obeum A2 162* |
| *Eubacterium rectale M104 1* | *Ruminococcus torques L2 14* |
| *Eubacterium siraeum 70 3* | *Sulfobacillus acidophilus NAL DSM 10332* |
| *Eubacterium siraeum V10Sc8a* | *Sulfobacillus acidophilus NAL DSM 10332* |
| *Faecalibacterium prausnitzii L2 6* | *Symbiobacterium thermophilum IAM 14863* |
| *Faecalibacterium prausnitzii SL3 3* | *Syntrophobotulus glycolicus FlGlyR DSM 8271* |
| *Finegoldia magna ATCC 29328* | *Syntrophomonas wolfei Goettingen DSM 2245B* |
| *Heliobacterium modesticaldum Ice1* | *Syntrophothermus lipocalidus DSM 12680* |
| *Pelotomaculum thermopropionicum SI* | *Thermaerobacter marianensis 7p75a DSM 12885* |
| **Bacteroidales (281 core genes, 126 CSCG)** | |
| *Alistipes shahii WAL 8301* | *Bacteroides salanitronis BL78 DSM 18170* |
| *Bacteroides coprosuis PC 139 DSM 18011* | *Bacteroides thetaiotaomicron VPI 5482* |
| *Bacteroides fragilis 638R* | *Bacteroides vulgatus ATCC 8482* |
| *Bacteroides fragilis NCTC 9343* | *Bacteroides xylanisolvens XB1A* |
| *Bacteroides fragilis YCH46* | *Barnesiella viscericola C46 DSM 18177* |
| *Bacteroides helcogenes P 36 108 DSM 20613* | *Candidatus Azobacteroides pseudotrichonymphae gv CFP2* |
| *Odoribacter splanchnicus 1651 6 DSM 20712* | *Porphyromonas gingivalis W83* |
| *Paludibacter propionicigenes WB4 DSM 17365* | *Prevotella intermedia 17* |
| *Parabacteroides distasonis ATCC 8503* | *Prevotella melaninogenica ATCC 25845* |

**Table S6.** continued

| **Bacteroidales, continued** | |
| --- | --- |
| *Porphyromonas asaccharolytica VPI 4198 DSM 20707* | *Prevotella multisaccharivorax PPPA20 DSM 17128* |
| *Porphyromonas gingivalis ATCC 33277* | *Prevotella ruminicola 23* |
| **Bacillales (456 core genes, 109 CSCG)** | |
| *Alicyclobacillus acidocaldarius 104 IA DSM 446* | *Bacillus cereus AH820* |
| *Anoxybacillus flavithermus WK1 DSM 2641* | *Bacillus cereus ATCC 10987* |
| *Bacillus amyloliquefaciens Campbell F DSM 7* | *Bacillus cereus ATCC 14579* |
| *Bacillus amyloliquefaciens FZB42* | *Bacillus cereus B4264* |
| *Bacillus anthracis A0248* | *Bacillus cereus E33L ZK* |
| *Bacillus anthracis Ames* | *Bacillus cereus G9842* |
| *Bacillus anthracis Ames Ancestor A2084* | *Bacillus cereus Q1* |
| *Bacillus anthracis CDC 684* | *Bacillus cereus cytotoxis NVH 391 98* |
| *Bacillus anthracis CI* | *Bacillus clausii KSM K16* |
| *Bacillus anthracis Sterne* | *Bacillus coagulans 36D1* |
| *Bacillus atrophaeus 1942* | *Bacillus halodurans C 125* |
| *Bacillus cellulosilyticus N 4 DSM 2522* | *Bacillus licheniformis DSM 13 Goettingen* |
| *Bacillus cereus 03BB102* | *Bacillus licheniformis DSM 13 Novozymes* |
| *Bacillus cereus AH187 F4810 72* | *Bacillus megaterium DSM 319* |
| *Bacillus megaterium QM B1551* | *Geobacillus sp Y412MC52* |
| *Bacillus pseudofirmus OF4* | *Geobacillus sp Y412MC61* |
| *Bacillus pumilus SAFR 032* | *Geobacillus thermodenitrificans NG80 2* |
| *Bacillus selenitireducens MLS10* | *Listeria innocua Clip11262* |
| *Bacillus subtilis BSn5* | *Listeria monocytogenes 08 5578* |
| *Bacillus subtilis natto BEST195* | *Listeria monocytogenes 08 5923* |

**Table S6.** continued

| **Bacillales, continued** | |
| --- | --- |
| *Bacillus subtilis spizizenii W23* | *Listeria monocytogenes 4a L99* |
| *Bacillus subtilis subtilis 168* | *Listeria monocytogenes 4b CLIP 80459* |
| *Bacillus thuringiensis Al Hakam* | *Listeria monocytogenes 4b F2365* |
| *Bacillus thuringiensis BMB171* | *Listeria monocytogenes EGD e* |
| *Bacillus thuringiensis sv konkukian 97 27* | *Listeria monocytogenes HCC23* |
| *Bacillus thuringiensis sv finitimus YBT 020* | *Listeria seeligeri sv 1 2b SLCC3954* |
| *Bacillus tusciae T2 DSM 2912* | *Listeria welshimeri sv 6b SLCC5334* |
| *Bacillus weihenstephanensis KBAB4* | *Lysinibacillus sphaericus C3 41* |
| *Brevibacillus brevis NBRC 100599* | *Macrococcus caseolyticus JCSC5402* |
| *Exiguobacterium sibiricum 255 15 DSM 17290* | *Oceanobacillus iheyensis HTE831* |
| *Exiguobacterium sp AT1b* | *Paenibacillus polymyxa E681* |
| *Geobacillus kaustophilus HTA426* | *Paenibacillus polymyxa SC2* |
| *Geobacillus sp C56 T3* | *Paenibacillus sp JDR 2* |
| *Geobacillus sp WCH70* | *Paenibacillus sp Y412MC10* |
| *Geobacillus sp Y4 1MC1* | *Staphylococcus aureus 04 02981* |
| *Staphylococcus aureus RF122* | *Staphylococcus aureus aureus Newman* |
| *Staphylococcus aureus aureus COL* | *Staphylococcus aureus aureus ST398* |
| *Staphylococcus aureus aureus ED133* | *Staphylococcus aureus aureus TW20 0582* |
| *Staphylococcus aureus aureus JH1* | *Staphylococcus aureus aureus USA300* |
| *Staphylococcus aureus aureus JH9* | *Staphylococcus aureus aureus USA300 TCH1516* |
| *Staphylococcus aureus aureus JKD6008* | *Staphylococcus aureus subsp aureus ED98* |
| *Staphylococcus aureus aureus JKD6159* | *Staphylococcus carnosus carnosus TM300* |
| *Staphylococcus aureus aureus M* | *Staphylococcus epidermidis ATCC 12228* |

**Table S6.** continued

| **Bacillales, continued** | |
| --- | --- |
| *Staphylococcus aureus aureus MRSA252* | *Staphylococcus epidermidis RP62A* |
| *Staphylococcus aureus aureus MSSA476* | *Staphylococcus haemolyticus JCSC1435* |
| *Staphylococcus aureus aureus MW2* | *Staphylococcus lugdunensis HKU09 01* |
| *Staphylococcus aureus aureus Mu3* | *Staphylococcus pseudintermedius ED99* |
| *Staphylococcus aureus aureus N315* | *Staphylococcus pseudintermedius HKU10 03* |
| *Staphylococcus aureus aureus NCTC 8325* | *Staphylococcus saprophyticus saprophyticus ATCC 15305* |
| *Catenulispora acidiphila ID139908 DSM 44928* | *Geodermatophilus obscurus DSM 43160* |
| *Cellulomonas flavigena 134 DSM 20109* | *Gordonia bronchialis DSM 43247* |
| *Clavibacter michiganensis michiganensis NCPPB 382* | *Intrasporangium calvum 7KIP DSM 43043* |
| *Clavibacter michiganensis sepedonicus ATCC 33113* | *Jonesia denitrificans 55134 DSM 20603* |
| *Corynebacterium aurimucosum CN 1 ATCC 700975* | *Kineococcus radiotolerans SRS30216* |
| *Corynebacterium diphtheriae gravis NCTC 13129* | *Kocuria rhizophila DC2201* |
| *Corynebacterium efficiens YS 314* | *Kribbella flavida DSM 17836* |
| *Corynebacterium glutamicum Kalinowski ATCC 13032* | *Kytococcus sedentarius 541 DSM 20547* |
| *Corynebacterium glutamicum Nakagawa ATCC 13032* | *Leifsonia xyli xyli CTCB07* |
| *Corynebacterium glutamicum R* | *Micrococcus luteus Fleming NCTC 2665* |
| *Corynebacterium jeikeium K411* | *Micromonospora aurantiaca ATCC 27029* |
| *Corynebacterium kroppenstedtii DSM 44385* | *Micromonospora sp L5* |
| *Corynebacterium pseudotuberculosis 1002* | *Mobiluncus curtisii ATCC 43063* |
| *Corynebacterium pseudotuberculosis C231* | *Mycobacterium abscessus CIP 104536* |

**Table S6.** continued

| *Corynebacterium pseudotuberculosis FRC41* | *Mycobacterium avium 104* |
| --- | --- |
| *Corynebacterium pseudotuberculosis I19* | *Mycobacterium avium paratuberculosis K 10* |
| *Corynebacterium urealyticum DSM 7109* | *Mycobacterium bovis AF2122 97* |
| *Frankia alni ACN14a* | *Mycobacterium bovis BCG Pasteur 1173P2* |
| *Frankia sp CcI3* | *Mycobacterium bovis BCG Tokyo 172* |
| *Frankia sp EAN1pec* | *Mycobacterium gilvum PYR GCK* |
| *Mycobacterium leprae Br4923* | *Propionibacterium freudenreichii shermanii CIRM BIA1* |
| *Mycobacterium leprae TN* | *Renibacterium salmoninarum ATCC 33209* |
| *Mycobacterium marinum M ATCC BAA 535* | *Rhodococcus equi 103S* |
| *Mycobacterium smegmatis MC2 155* | *Rhodococcus erythropolis PR4* |
| *Mycobacterium sp JLS* | *Rhodococcus jostii RHA1* |
| *Mycobacterium sp KMS* | *Rhodococcus opacus B4* |
| *Mycobacterium sp MCS* | *Rothia dentocariosa ATCC 17931* |
| *Mycobacterium tuberculosis CDC1551* | *Rothia mucilaginosa DY 18* |
| *Mycobacterium tuberculosis F11 ExPEC* | *Saccharomonospora viridis P101 DSM 43017* |
| *Mycobacterium tuberculosis H37Ra* | *Saccharopolyspora erythraea NRRL 2338 white* |
| *Mycobacterium tuberculosis H37Rv lab strain* | *Salinispora arenicola CNS 205* |
| *Mycobacterium tuberculosis KZN 1435 MDR* | *Salinispora tropica CNB 440* |
| *Mycobacterium ulcerans Agy99* | *Sanguibacter keddieii ST 74 DSM 10542* |
| *Mycobacterium vanbaalenii PYR 1* | *Segniliparus rotundus CDC 1076 DSM 44985* |
| *Nakamurella multipartita Y 104 DSM 44233* | *Stackebrandtia nassauensis LLR 40K 21 DSM 44728* |
| *Nocardia farcinica IFM 10152* | *Streptomyces avermitilis MA 4680* |
| *Nocardioides sp JS614* | *Streptomyces bingchenggensis BCW 1* |
| *Nocardiopsis dassonvillei dassonvillei DSM 43111* | *Streptomyces coelicolor A3 2* |

**Table S6.** continued

| **Actinomycetales, continued** | |
| --- | --- |
| *Propionibacterium acnes KPA171202* | *Streptomyces griseus griseus NBRC 13350* |
| *Propionibacterium acnes SK137* | *Streptomyces scabiei 87 22* |
| *Streptosporangium roseum DSM 43021* | *Tropheryma whipplei TW08 27* |
| *Thermobifida fusca YX* | *Tropheryma whipplei Twist* |
| *Thermobispora bispora R51 DSM 43833* | *Tsukamurella paurometabola 33 DSM 20162* |
| *Thermomonospora curvata DSM 43183* | *Xylanimonas cellulosilytica DSM 15894* |
| **Thermoanaerobacterales (408 core gene, 186 CSCG)** | |
| *Ammonifex degensii KC4* | *Moorella thermoacetica ATCC 39073* |
| *Caldicellulosiruptor bescii Z 1320 DSM 6725* | *Thermanaeromonas toyohensis ToBE DSM 14490* |
| *Caldicellulosiruptor hydrothermalis 108* | *Thermoanaerobacter brockii finnii Ako 1 DSM 3389* |
| *Caldicellulosiruptor kristjanssonii 177R1B DSM 12137* | *Thermoanaerobacter italicus Ab9 DSM 9252* |
| *Caldicellulosiruptor kronotskyensis 2002* | *Thermoanaerobacter mathranii mathranii A3 DSM11426* |
| *Caldicellulosiruptor obsidiansis OB47* | *Thermoanaerobacter pseudethanolicus 39E ATCC 33223* |
| *Caldicellulosiruptor owensensis OL* | *Thermoanaerobacter sp X513* |
| *Caldicellulosiruptor saccharolyticus DSM 8903* | *Thermoanaerobacter sp X514* |
| *Carboxydothermus hydrogenoformans Z 2901* | *Thermoanaerobacter tengcongensis MB4T* |
| *Coprothermobacter proteolyticus DSM 5265* | *Thermoanaerobacterium thermosaccharolyticum DSM 571* |
| *Mahella australiensis 50 1 BON DSM 15567* | *Thermodesulfobium narugense Na82 DSM 14796* |
